# KRAS G12C inhibitors in KRAS^G12C^-mutated solid tumors: an immunologically informed systematic review and reconstructed individual patient data meta-analysis

**DOI:** 10.3389/fimmu.2026.1848431

**Published:** 2026-07-10

**Authors:** Yici Yan, Leyi Zheng, Hongfei Wang, Leitao Sun, Xing Xu

**Affiliations:** 1 The First Affiliated Hospital of Zhejiang Chinese Medical University (Zhejiang Provincial Hospital of Chinese Medicine), Hangzhou, China; 2 Academy of Chinese Medical Science, Zhejiang Chinese Medical University, Hangzhou, China; 3 Key Laboratory of Neuropharmacology and Translational Medicine of Zhejiang Province, School of Pharmaceutical Sciences, Zhejiang Chinese Medical University, Hangzhou, China; 4 Pinghu Traditional Chinese Medicine Hospital, JiaXing, China

**Keywords:** efficacy, immunology, IPD meta-analysis, KRAS G12C inhibitor, PD-L1, solid tumors

## Abstract

**Background:**

Despite the proven efficacy of KRAS G12C inhibitors (KRAS G12Ci) in solid tumors, evidence from direct comparisons with standard of care is scarce, and no analysis has investigated the potential immunological basis for differential responses.

**Methods:**

PubMed, Embase, Cochrane Library, and ClinicalTrials.gov for randomized controlled trials (RCTs) involving solid tumors patients who had received KRAS G12Ci were retrieved from inception to March 28, 2026. Individual participant data on progression-free survival (PFS) and overall survival (OS) were extracted from the published Kaplan-Meier survival curves. When available, subgroup data by programmed death ligand 1 (PD-L1) expression were extracted to explore immune-related correlates of treatment response.

**Results:**

A total of 4 articles with 3 RCTs and 949 participants were selected. In 1-stage reconstructed individual patient data meta-analyses, PFS was better in the KRAS G12Ci group (HR, 0.62; 95% CI, 0.53-0.74; *P* < 0.001). However, no statistical difference in OS was observed (HR, 0.93; 95% CI, 0.74-1.16; *P* = 0.495). The results were confirmed by 2-stage meta-analyses which additionally exhibited an objective response rate (ORR) of 3.60 (95% CI; 2.01-6.46; *P* < 0.001; I^2^ = 39.7%). Regarding PD-L1 expression, PFS benefits were observed in patients with expression levels <1% (HR, 0.56; 95% CI: 0.38-0.83; *P* = 0.004) and 1%-49% (HR, 0.58; 95% CI: 0.43-0.78; *P* < 0.001). KRAS G12Ci demonstrated a better safety profile, apart from diarrhea and rash.

**Conclusions:**

A similar OS, but better PFS and ORR with a superior safety profile were observed in patients receiving KRAS G12Ci, suggesting that KRAS G12Ci may be more suitable for later-line therapy. Older patients, those without liver metastases, or those with PD-L1<50% may be the target population. These subgroup observations are hypothesis-generating and require prospective validation.

**Systematic Review Registration:**

https://www.crd.york.ac.uk/PROSPERO/, identifier CRD420251146769.

## Introduction

Kirsten rat sarcoma viral oncogene homologue (KRAS) is the most common oncogenic driver across human cancer (1, 2). Among its various mutations, the Glycine-to-Cysteine mutation at position 12 (G12C) variant is of particular clinical significance, accounting for roughly 13-15% of non-small cell lung cancer (NSCLC) and 3-4% of colorectal cancer (CRC) cases (3–5). The KRAS G12C mutation has conferred a worse prognosis in solid tumors by reshaping the tumor immune microenvironment (TIME) (6). Oncogenic KRAS signaling has been shown to upregulate Programmed Cell Death Ligand 1 (PD-L1) expression (7), recruit immunosuppressive cells such as myeloid-derived suppressor cells (MDSCs), and impair the function of tumor-infiltrating T lymphocytes, collectively fostering an immune-evasive landscape (8, 9). Compounding this immunological challenge, directly targeting KRAS G12C mutant protein proved exceptionally difficult, as its structure offered no deep binding pockets for inhibitors and its strong binding to GTP resisted drug discovery efforts (10).

However, the groundbreaking discovery of a druggable “switch-II pocket” in KRAS by Ostrem et al (11). in 2013 has paved the way for the development of clinically active KRAS inhibitors. Only 8 years later, sotorasib, the first direct KRAS G12C inhibitor (KRAS G12Ci) targeting this inducible allosteric pocket gained accelerated approval by the Food and Drug Administration (FDA) based on the finding from the CodeBreak 100 trial, where sotorasib achieved an objective response rate (ORR) of 37.1% and a median progression-free survival (mPFS) of 6.8 months (12). This milestone was rapidly followed by the accelerated approval of adagrasib, fulzerasib and garsorasib, which demonstrated a similar profile with an ORR of 42.9%, 49.1% and 50%, respectively, in NSCLC (13–15). Combination regimens involving KRAS G12Ci have also demonstrated encouraging efficacy. Based on results from the CodeBreaK 300 (16) and KRYSTAL-1 trials (17), the FDA approved adagrasib plus cetuximab for CRC in 2024, followed by sotorasib plus panitumumab in 2025. Despite these advances in KRAS G12C-targeted therapy for NSCLC and CRC, clinical guidelines from the National Comprehensive Cancer Network (NCCN) and the Chinese Society of Clinical Oncology (CSCO) have not yet aligned with this progress in their recommendations for KRAS G12Ci monotherapy in CRC. Furthermore, the Phase 3 CodeBreak 200 trial indicated that sotorasib did not improve overall survival (OS), raising concerns about its long-term efficacy and highlighting the necessity to conduct a comprehensive and quantitative meta-analysis (18).

Beyond these clinical uncertainties, acquired resistance remains a critical bottleneck for KRAS G12Ci (19). Accumulating evidence has suggested that TIME may play a key role in this process (20, 21). In particular, PD-L1 upregulation in KRAS G12C-mutant tumors has been shown to drive immunosuppression and contribute to acquired resistance (22, 23). Given the emerging intersection between KRAS inhibition and the TIME, we sought to not only evaluate the overall efficacy and safety of KRAS G12Ci, but also explore whether PD-L1 expression, a key immune biomarker, influences treatment response. Therefore, we conducted a constructed individual patient data (IPD) meta-analysis of randomized controlled trials (RCTs) to definitively assess the efficacy and safety of KRAS G12Ci in solid tumors, with a particular focus on exploring the potential role of PD-L1 expression in treatment response.

## Methods

### Literature search strategy

This meta-analysis was performed in adherence to the Preferred Reporting Items for Systematic Reviews and Meta-Analyses (PRISMA) guidelines. Two investigators independently conducted a systematic search of PubMed, Embase, and Cochrane Library from inception to March 28, 2026, using the MeSH terms and keywords “KRAS G12Ci” and “Neoplasms” (**eTable 1**). The search was supplemented by screening ClinicalTrials.gov, American Society of Clinical Oncology (ASCO), European Society for Medical Oncology (ESMO) and Reference lists of included studies. The search was limited to English publications but no restrictions on article type were applied to maximize coverage. The protocol was preregistered in the International Prospective Register of Systematic Reviews (CRD420251146769).

### Inclusion and exclusion criteria

The inclusion criteria were as follows (1): patients with solid tumors (2); intervention with an FDA-approved KRAS G12Ci, alone or in combination (3); outcomes comprising OS or progression-free survival (PFS), with or without ORR and adverse events (AEs) (4); RCTs.

The exclusion criteria were as follows (1): control arms that involved a KRAS G12Ci (2); single-arm trials, reviews, observational studies, case reports, letters, and commentaries.

The inclusion and exclusion criteria were established by two investigators, with any discrepancies resolved through consensus involving a third reviewer.

### Assessment of bias and the overall quality of evidence

The risk of bias in the included RCTs was independently assessed by two authors using the Cochrane risk-of-bias tool, and were visualized using Review Manager 5.4. The Grading of Recommendations Assessment, Development, and Evaluation (GRADE) framework was subsequently applied to evaluate the certainty of the evidence for each outcome.

### Data extraction

Two investigators independently extracted the data and carried out cross-checks (1): study characteristic: first author, publication year, trial name, line, histology, regimen, the number of patients, design, masking, phase, stage, median follow-up, median age (2); study outcomes: effect estimates of OS, PFS, ORR, and AEs of all grade and ≥grade 3; OS was defined as the time from randomization to death from any cause. PFS was defined as the time from randomization to the first documented disease progression or death from any cause (3). subgroup data: when available, we extracted subgroup estimates stratified by PD-L1 expression level to explore the potential immunological implications of KRAS G12Ci, given the established role of PD-L1 in mediating immune evasion in KRAS-mutant tumors.

The IPD information for PFS and OS were extracted from the published Kaplan-Meier (K-M) curves using Engauge Digitizer, version 12.1. For each study, the survival curves were reconstructed, and the accuracy of this process was validated using the method described by Liu N et al (24). The hazard ratios (HRs) and 95% confidence intervals (CIs) between the KRAS G12Ci and the control group were then calculated employing a Cox regression model. The high accuracy of the reconstructed IPD was confirmed by low error metrics, including a root mean square error (RMSE) of <0.05, a mean absolute error of <0.02, and a maximum absolute error of <0.05. In cases where these accuracy thresholds were not met, the data extraction process would be repeated.

### One-stage meta-analysis

Reconstructed K-M curves for PFS and OS of the entire study population were generated from the pooled IPD. In the one-stage meta-analysis, a Cox proportional hazards model with trial specified as the random effect, was employed to calculate the HRs and 95% CIs between the KRAS G12Ci and the control group. Heterogeneity was evaluated from the between-study variance estimate of this model (21).

### Two-stage meta-analysis

All statistical analyses were performed using Stata 18.0. Given the anticipated clinical heterogeneity in tumor types and treatment regimens, a random-effects model was applied. Heterogeneity was quantified using the Chi-square Q test and I² statistic, with values of <30%, 30-60%, and >60% indicating low, moderate, and high heterogeneity, respectively. Effect sizes are presented as HR for PFS and OS, and relative risks (RR) for ORR, AEs, each with 95% CIs. We conducted sensitivity analyses, including leave-one-out analyses and subgroup-restricted analyses, to assess the robustness of our findings. Publication bias was not performed given the small number of included studies. All p-values were two-tailed, with statistical significance defined as *P* < 0.05.

## Results

### Study selection and study characteristics

**Figure 1:**
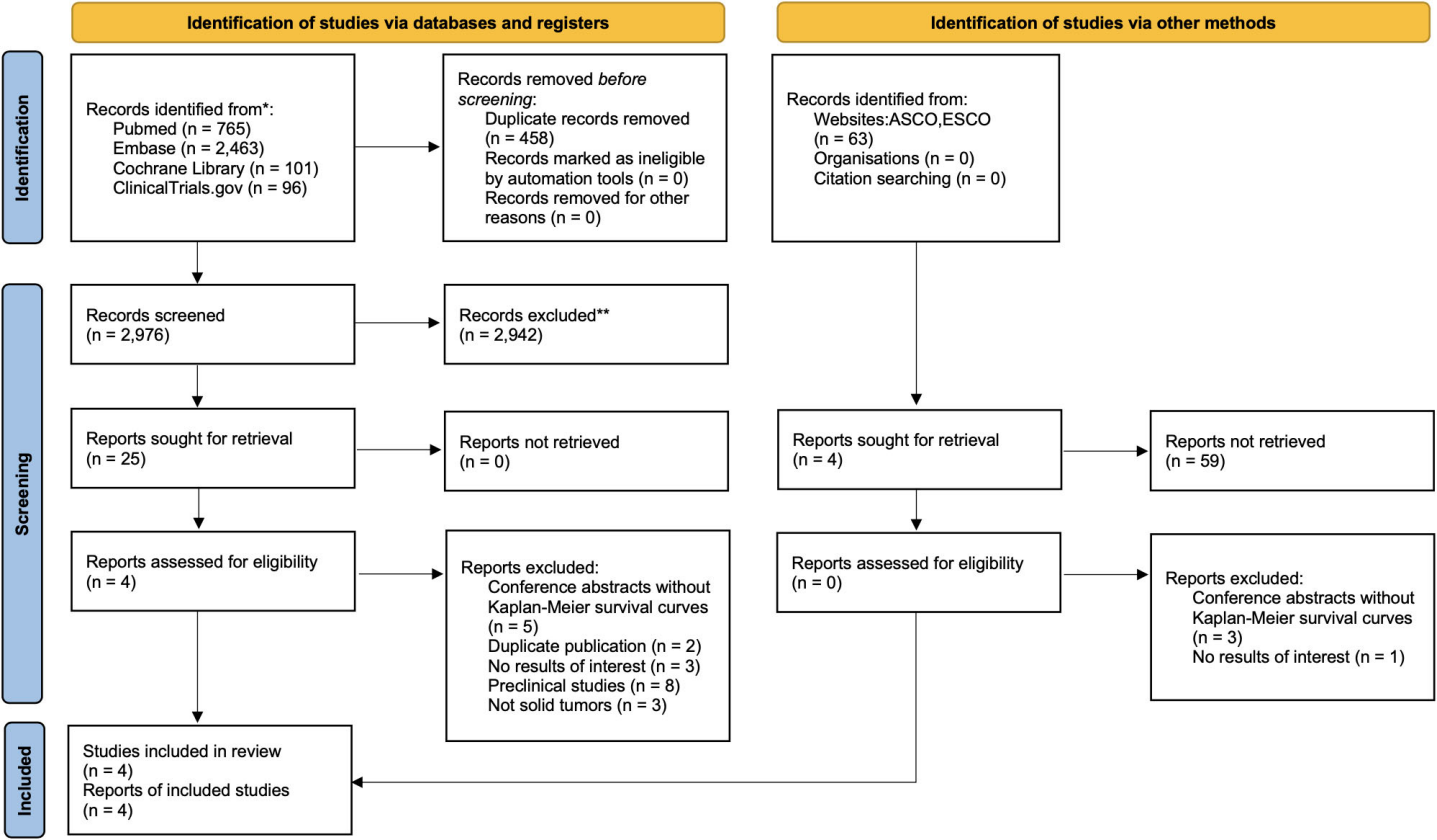
Flowchart of the study selection.

Literature searches resulted in 3,488 potentially relevant citations. After eliminating duplicates and excluding records by reading titles and abstracts, 25 articles remained. Regarding report exclusion after full-text review, 5 were conference abstracts without K-M survival curves, 2 were duplicate publications, 3 lacked results of interest, 8 were preclinical studies and 3 were not solid tumors. Eventually, 4 studies (16, 18, 25, 26) with 3 RCTs were included in this review. The detailed selection process is depicted in **Figure 1**.

All the studies were phase 3 multicenter RCTs with open-label design. The mean age of patients ranged between 58 and 65 years and the median follow-up varied from 7.2 and 17.7 months. From the four studies, 789 patients with KRAS G12C-mutated NSCLC and 160 patients with KRAS G12C-mutated metastatic CRC were included in the meta-analysis of ORR, OS and PFS. Notably, the studies by Fakih (16) and Pietrantonio (25) derived from the same clinical trial (CodeBreaK 300). Both were included as they reported partial survival data: Fakih provided PFS but no OS, while Pietrantonio reported OS but lacked PFS. All studies were intended for patients who had undergone more than one prior line of therapy. The therapeutic regimens applied in the studies mainly fell into two categories: monotherapy therapy and combination therapy. The monotherapy options included 600 mg adagrasib and 960 mg sotorasib. The combination consisted of 960 mg sotorasib+6 mg/kg panitumumab, and 240 mg sotorasib+6 mg/kg panitumumab. For the control groups, the regimens administered were 75 mg/m² docetaxel, 35 mg/m² rifluridine-tipiracil, or 160 mg regorafenib. All three trials included in our analysis used RECIST 1.1 criteria for ORR assessment. Detailed information regarding the included studies is presented in **Table 1** and **eTable 2**.

**Table 1:** Characteristic of included studies.

Trial (NCT)	Source	Line	Histology	Regimen	No. of patients	Median PFS, (95% CI),mo	Median OS (95% CI),mo
CodeBreak 200 (NCT04303780)	Langen, 2023	>1	KRAS G12C- mutated NSCLC	I: 960 mg Sotorasib C: 75 mg/m^2^ Docetaxel	171	5.6 (4.3-7.8)	10.6 (8.9-14.0)
					174	4.5 (3.0-5.7)	11.3 (9.0-14.9)
CodeBreaK 300 (NCT05198934)	Fakih, 2023	>1	KRAS G12C-mutated mCRC	Ia: 960 mg Sotorasib+6 mg/kg Panitumumab Ib: 240mg Sotorasib+6 mg/kg Panitumumab C: 35 mg/m^2^ Trifluridine-tipiracil or 160 mg Regorafenib	53	5.6 (4.2-6.3)	NE
					53	3.9 (3.6-5.7)	NE
					54	2.0 (1.9-3.9)	NE
CodeBreaK 300 (NCT05198934)	Pietrantonio, 2025	>1	KRAS G12C-mutated mCRC	Ia: 960 mg Sotorasib+6 mg/kg Panitumumab Ib: 240mg Sotorasib+6 mg/kg Panitumumab C: 35 mg/m^2^ Trifluridine-tipiracil or 160 mg Regorafenib	53	NE	NE (8.6-NE)
					53	NE	11.9 (7.5-NE)
					54	NE	10.3 (7.0-NE)
KRYSTAL 12 (NCT04685135)	Barlesi, 2025	>1	KRAS G12C- mutated NSCLC	I: 600mg Adagrasib C: 75 mg/m² Docetaxel	301	5.5 (4.5-6.7)	NE
					152	3.8 (2.7-4.7)	NE

KRAS, kirsten rat sarcoma viral oncogene homolog; G12C, Glycine 12 Cysteine; NSCLC, non-small cell lung cancer; mCRC, metastatic colorectal Cancer; I, intervention group; C, control group; No, numbers; PFS, progression-free survival; OS, overall survival; CI, Confidence Interval; mo, month; NE, not estimable.

### Reconstructed survival curves

**Figure 2:**
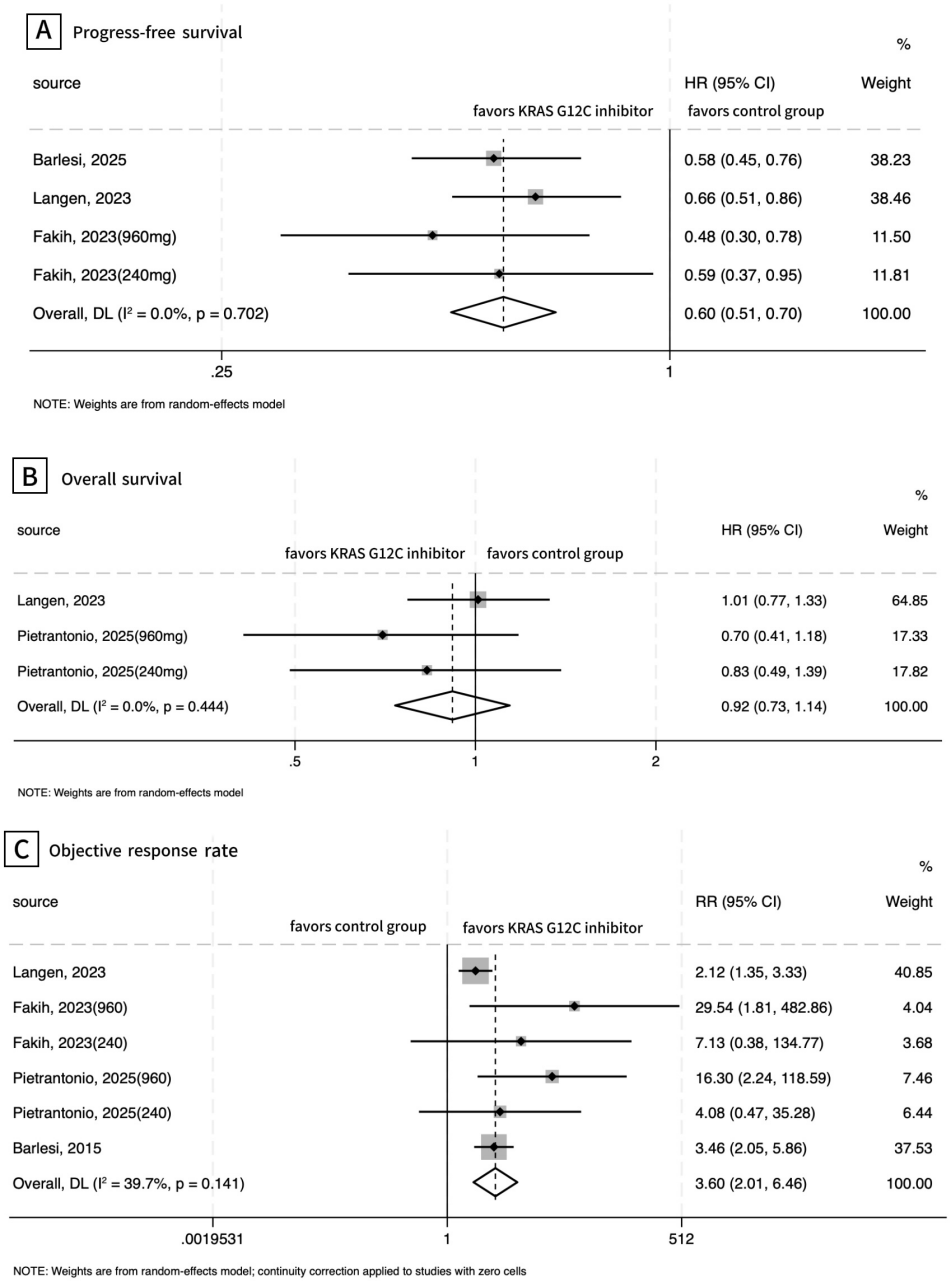
Reconstructed Kaplan-Meier survival curves and 1-stage meta-analysis. Reconstructed Kaplan-Meier survival curves and 1-stage meta-analysis of PFS **(A)**, OS **(B)**, and ORR **(C)**.

All reconstructed K-M curves showed high consistency with the published K-M curves of each included study, and the difference between estimated and read-in survival probabilities was negligible (**eAppendix 1**). The reconstructed survival curves of PFS and OS for the combined population are presented in **Figures 2A, B**. At 12 months, the estimated PFS rates were 23.3% (95% CI, 18.9%-28.7%) for the KRAS G12Ci group and 8.3% (95% CI, 4.9%-14.1%) for the control group. The estimated 12-months OS rates were 47.7% (95% CI, 42.0%-54.2%) for the KRAS G12Ci group and 46.1% (95% CI, 40.2%-52.8%) for the control group.

### One-stage meta-analysis

In one-stage IPD meta-analysis, the Cox-based model was used to calculate the HR. The KRAS G12Ci group exhibited PFS advantage over the control group, with a HR of 0.62 (95% CI, 0.53-0.74; *P* < 0.001) (**Figure 2A**). However, there was no statistical difference in OS between the two groups (HR, 0.93; 95% CI, 0.74-1.16; *P* = 0.495) (**Figure 2B**).

### Two-stage meta-analysis

**Figure 3:**
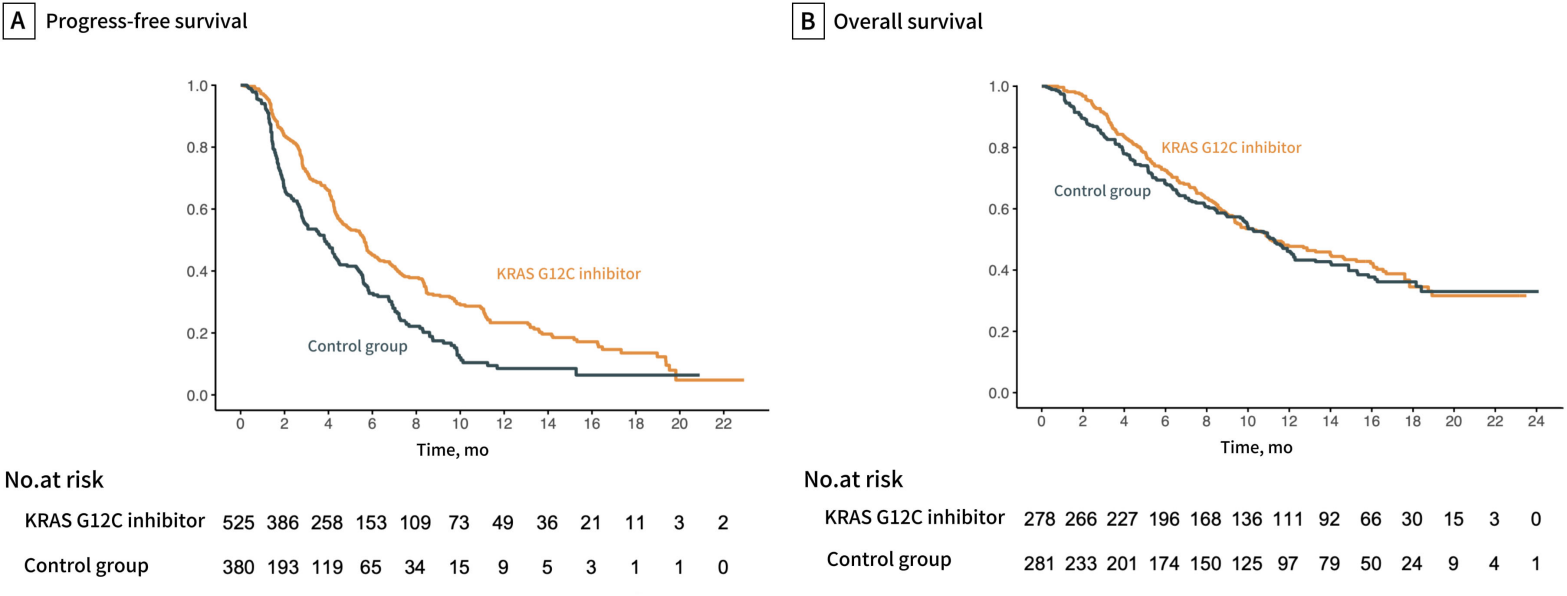
Reconstructed Kaplan-Meier survival curves and 1-stage meta-analysis of PFS **(A)** and OS **(B)**.

Two-stage study-level meta-analysis was conducted to validate results robustness. The pooled HR for PFS was 0.60 (95% CI, 0.51-0.70; *P* < 0.001), with negligible heterogeneity at I^2^ = 0.0% (**Figure 3A**). For OS, the pooled HR was 0.92 (95% CI, 0.73-1.14; *P* = 0.430), with negligible heterogeneity (I^2^ = 0.0%) (**Figure 3B**). Both HRs of PFS and OS were similar to the HRs of the 1-stage analysis. For ORR, the pooled RR was 3.60 (95% CI, 2.01-6.46; *P* < 0.001), with moderate heterogeneity (I^2^ = 39.7%) (**Figure 3C**).

### Immune-related subgroup analysis

Given the emerging role of the tumor immune microenvironment in KRAS G12Ci response, we performed a subgroup analysis by PD-L1 expression level where data were available. PD-L1 stratified outcomes were reported only in the two NSCLC trials (18, 25), both of which evaluated KRAS G12C inhibitor monotherapy. The CRC trial (16, 26) which tested sotorasib in combination with panitumumab, did not report PD-L1 stratified outcomes. As shown in **Table 2**, PFS benefits were observed in patients with expression levels <1% (HR = 0.56, 95% CI: 0.38-0.83; *P* = 0.004) and 1%-49% (HR = 0.58, 95% CI: 0.43-0.78; *P* < 0.001). In the ≥50% subgroup, the PFS improvement was of marginal significance and did not reach the statistical threshold (HR = 0.68, 95% CI: 0.47-1.01; *P* = 0.054). Although the global interaction test across PD-L1 subgroups was not statistically significant (P = 0.730, **eTable 3**), the observed benefit was confined to the PD-L1 <50% subgroups, indicating that potential treatment effects may be more pronounced in patients with lower PD-L1 expression.

**Table 2:** Subgroup analysis of PFS and OS.

Subgroup	PFS			OS		
	No. of studies	HR (95% CI)	p	No. of studies	HR (95% CI)	p
Age						
<65 yrs	4	0.61 (0.49, 0.75)	<0.001	2	1.23 (0.76, 2.00)	0.398
≥65 yrs	4	0.57 (0.43, 0.74)	<0.001	2	0.36 (0.18, 0.73)	0.005
Body site						
Colon	2	0.49 (0.32, 0.74)	0.001	2	0.80 (0.50, 1.27)	0.343
Rectum	2	0.51 (0.29, 0.92)	0.024	2	0.68 (0.29, 1.61)	0.380
Bone metastasis						
Yes	2	0.61 (0.45, 0.82)	0.001	NA	NA	NA
No	2	0.58 (0.46, 0.74)	<0.001	NA	NA	NA
ECOG score						
0	2	0.53 (0.37, 0.76)	0.001	NA	NA	NA
1	2	0.61 (0.49, 0.76)	<0.001	NA	NA	NA
Histology						
NSCLC	2	0.62 (0.51, 0.74)	<0.001	NA	NA	NA
CRC	2	0.53 (0.38, 0.75)	<0.001	NA	NA	NA
Previous line						
1 or 2	2	0.47 (0.31, 0.72)	0.001	2	0.82 (0.50, 1.33)	0.418
≥3	2	0.58 (0.32, 1.05)	0.074	2	0.81 (0.47, 1.42)	0.465
Liver metastasis						
Yes	4	0.43 (0.32, 0.57)	<0.001	2	0.80 (0.52, 1.23)	0.319
No	4	0.63 (0.52, 0.76)	<0.001	2	0.40 (0.17, 0.95)	0.039
Location of tumor						
Right side	2	0.49 (0.28, 0.85)	0.012	2	0.74 (0.39, 1.39)	0.346
Left side	2	0.60 (0.39, 0.92)	0.019	2	0.86 (0.53, 1.37)	0.514
PD-L1 expression						
<1%	2	0.56 (0.38, 0.83)	0.004	NA	NA	NA
1%-49%	2	0.58 (0.43, 0.78)	<0.001	NA	NA	NA
≥50%	2	0.68 (0.47, 1.01)	0.054	NA	NA	NA
Race						
Asian	2	0.51 (0.27, 0.99)	0.046	NA	NA	NA
Non-Asian	2	0.63 (0.49, 0.81)	<0.001	NA	NA	NA
Regimen type						
monotherapy	2	0.62 (0.51, 0.74)	<0.001	NA	NA	NA
combination	2	0.53 (0.38, 0.75)	<0.001	NA	NA	NA
Sex						
Female	4	0.60 (0.46, 0.80)	<0.001	2	0.87 (0.52, 1.46)	0.596
Male	4	0.57 (0.46, 0.70)	<0.001	2	0.65 (0.38, 1.10)	0.106

yrs, years; ECOG, Eastern Cooperative Oncology Group; NSCLC, non-small cell lung cancer; CRC, colorectal cancer; PD-L1, Programmed Death Ligand 1; PFS, progression-free survival; No, number; HR, hazard ratio; CI, confidence interval; OS, overall survival; NA, not available.

### Other subgroup analysis

Subgroup analysis was also conducted to explore potential variations in the treatment benefit of the KRAS G12Ci by age, body site, tumor location, bone metastasis, Eastern Cooperative Oncology Group (ECOG) score, histology, treatment line, liver metastasis, race, regimen type, and sex (**Table 2**).

The KRAS G12Ci conferred a PFS benefit in both patients <65 (HR = 0.61, 95% CI: 0.49-0.75; *P* < 0.001) and ≥65 years (HR = 0.57, 95% CI: 0.43-0.74; *P* < 0.001), but it improved OS only in those aged ≥65 years (HR = 0.36, 95% CI: 0.18-0.73; *P* = 0.005), with no significant advantage in patients <65 years (*P* = 0.398). Among patients with CRC, the KRAS G12Ci improved PFS in patients with colon (HR = 0.49, 95% CI: 0.32-0.74; *P* = 0.001) and rectal cancer (HR = 0.51, 95% CI: 0.29-0.92; *P* = 0.024). However, the treatment did not yield a statistically significant improvement in OS for either anatomic subgroup (colon, *P* = 0.343; rectal, *P* = 0.380). Both right-sided (HR = 0.49, 95% CI: 0.28-0.85; *P* = 0.012) and left-sided tumor patients (HR = 0.60, 95% CI: 0.39-0.92; *P* = 0.019) achieved PFS benefits with the intervention, yet OS advantages were of no statistical significance in either right-sided (*P* = 0.346) or left-sided tumor (*P* = 0.514) subgroups. For bone metastasis status, PFS prolongation was seen in the intervention group among patients with bone metastasis (HR = 0.61, 95% CI: 0.45-0.82; *P* = 0.001) and without bone metastasis (HR = 0.58, 95% CI: 0.46-0.74; *P* < 0.001). Regarding ECOG score, PFS benefits were observed in the intervention group for both ECOG score=0 (HR = 0.53, 95% CI: 0.37-0.76; *P* = 0.001) and ECOG score=1 (HR = 0.61, 95% CI: 0.49-0.76; *P* < 0.001). By histology, KRAS G12Ci improved PFS in NSCLC (HR = 0.62, 95% CI: 0.51-0.74; *P* < 0.001) and CRC (HR = 0.53, 95% CI: 0.38-0.75; *P* < 0.001) patients. When stratified by treatment line, patients receiving 1 or 2 lines of therapy showed PFS benefit from the intervention (HR = 0.47, 95% CI: 0.31-0.72; *P* = 0.001), while those with ≥3 lines had no statistically significant PFS improvement (*P* = 0.074). No significant OS benefit was observed in either subgroup (1–2 lines, *P* = 0.418; ≥3 lines, *P* = 0.465). Analysis by liver metastasis status revealed a PFS benefit in patients with liver metastases (HR = 0.43, 95% CI: 0.32-0.57; *P* < 0.001) as well as in those without liver metastases (HR = 0.63, 95% CI: 0.52-0.76; *P* < 0.001). An OS benefit was limited to patients without liver metastases (HR = 0.40, 95% CI: 0.17-0.95; *P* = 0.039), with no significant advantage observed in those with metastases (*P* = 0.319). For race, non-Asian (HR = 0.63, 95% CI: 0.49-0.81; *P* < 0.001) patients gained significant PFS benefits from the intervention. In Asian subgroup, the PFS improvement from the intervention was of marginal significance (HR = 0.51, 95% CI: 0.27-0.99; *P* = 0.046). As for regimen type, both monotherapy (HR = 0.62, 95% CI: 0.51-0.74; *P* < 0.001) and combination therapy subgroups (HR = 0.53, 95% CI: 0.38-0.75; *P* < 0.001) demonstrated PFS improvements. In terms of sex, the intervention improved PFS in both female (HR = 0.60, 95% CI: 0.46-0.80; *P* < 0.001) and male (HR = 0.57, 95% CI: 0.46-0.70; *P* < 0.001) patients, but OS benefits were of no statistical significance in either female (*P* = 0.596) or male (*P* = 0.106) subgroups.

### Safety

Given the substantial heterogeneity in control regimens across the included studies, safety analyses were stratified by comparator to provide clinically interpretable results (**Table 3**). Pooled safety analyses across all studies, which did not account for heterogeneity in control regimens, are presented in **eTable 4** for exploratory purposes.

**Table 3:** Treatment-related common adverse events stratified by comparison in this meta-analysis.

Adverse events		Comparison	No. of Studies	RR (95% CI)			
				All Grade	P value	Grade≥3	P value
Digestive system	Constipation	Docetaxel	1	NA	NA	NA	NA
		TAS-102 or Rego	2	1.43 (0.41, 5.02)	0.570	NA	NA
	Decreased appetite	Docetaxel	2	0.99 (0.72, 1.36)	0.940	NA	NA
		TAS-102 or Rego	2	0.56 (0.23, 1.38)	0.210	NA	NA
	Diarrhoea	Docetaxel	2	1.76 (1.40, 2.19)	<0.001	NA	NA
		TAS-102 or Rego	2	0.96 (0.57, 1.63)	0.890	5.72 (0.70, 46.88)	0.100
	Nausea	Docetaxel	2	1.39 (0.39, 5.01)	0.620	NA	NA
		TAS-102 or Rego	2	0.54 (0.28, 1.06)	0.070	1.43 (0.24, 8.61)	0.700
	Vomiting	Docetaxel	2	2.01 (0.27, 15.00)	0.500	NA	NA
		TAS-102 or Rego	2	0.98 (0.42, 2.28)	0.950	NA	NA
Hematological system	Anaemia	Docetaxel	2	0.42 (0.07, 2.51)	0.340	NA	NA
		TAS-102 or Rego	2	0.43 (0.21, 0.91)	0.030	0.37 (0.10, 1.38)	0.140
	Leukopenia	Docetaxel	1	NA	NA	NA	NA
		TAS-102 or Rego	2	0.11 (0.01, 0.83)	0.030	NA	NA
	Neutropenia	Docetaxel	2	0.19 (0.09, 0.39)	<0.001	NA	NA
		TAS-102 or Rego	2	0.03 (0.00, 0.21)	<0.001	0.04 (0.01, 0.28)	0.001
	Thrombocytopenia	Docetaxel	1	NA	NA	NA	NA
		TAS-102 or Rego	2	0.49 (0.12, 1.95)	0.310	NA	NA
Skin	Alopecia	Docetaxel	2	0.04 (0.01, 0.11)	<0.001	NA	NA
		TAS-102 or Rego	2	0.43 (0.10, 1.93)	0.270	NA	NA
	Rash	Docetaxel	1	NA	NA	NA	NA
		TAS-102 or Rego	2	14.43 (3.54, 58.84)	<0.001	6.74 (0.84, 53.84)	0.070
Others	Asthenia	Docetaxel	2	0.60 (0.36, 0.98)	0.040	NA	NA
		TAS-102 or Rego	2	0.48 (0.20, 1.15)	0.100	NA	NA
	Fatigue	Docetaxel	2	0.54 (0.13, 2.27)	0.400	NA	NA
	TAS-102 or Rego	2	0.43 (0.19, 0.94)	0.030	0.19 (0.02, 1.62)	0.130

Pani, Panitumumab; TAS-102, Trifluridine-tipiracil; Rego, Regorafenib; No, number; NA, not available.

In digestive system, KRAS G12Ci was associated with a higher risk of all-grade diarrhea (RR = 1.76, 95% CI: 1.40-2.19; P<0.001) compared with docetaxel. However, no significant difference in all-grade diarrhea was observed when compared with trifluridine-tipiracil or regorafenib (P = 0.890). For other digestive adverse events, no statistically significant differences were identified in either comparison: constipation (P = 0.570 for trifluridine-tipiracil or regorafenib), decreased appetite (P = 0.940 for docetaxel; P = 0.210 for trifluridine-tipiracil or regorafenib), nausea (P = 0.620 for docetaxel; P = 0.070 for trifluridine-tipiracil or regorafenib), and vomiting (P = 0.500 for docetaxel; P = 0.950 for trifluridine-tipiracil or regorafenib). For grade ≥3 digestive adverse events, no statistically significant differences were observed: diarrhea (P = 0.890 for trifluridine-tipiracil or regorafenib) and nausea (P = 0.700 for trifluridine-tipiracil or regorafenib). As for hematological toxicities, KRAS G12Ci was associated with lower risks of all-grade neutropenia (RR = 0.19, 95% CI: 0.09-0.39; P<0.001) and all-grade alopecia (RR = 0.04, 95% CI: 0.01-0.11; P<0.001) compared with docetaxel. In comparisons against trifluridine-tipiracil or regorafenib, KRAS G12Ci similarly demonstrated lower risks of neutropenia (RR = 0.03, 95% CI: 0.00-0.21; P<0.001) and leukopenia (RR = 0.11, 95% CI: 0.01-0.83; P = 0.030). No significant differences were observed for all-grade anemia (P = 0.340 for docetaxel; P = 0.030 for trifluridine-tipiracil or regorafenib) or grade ≥3 anemia (P = 0.140 for trifluridine-tipiracil or regorafenib). For grade ≥3 neutropenia, lower risk was observed in comparisons against trifluridine-tipiracil or regorafenib (RR = 0.04, 95% CI: 0.01-0.28; P = 0.001). Regarding skin-related AEs, KRAS G12Ci was associated with a significantly higher risk of all-grade rash (RR = 14.43, 95% CI: 3.54-58.84; P<0.001) compared with trifluridine-tipiracil or regorafenib. No significant differences were observed for grade ≥3 rash in comparisons against trifluridine-tipiracil or regorafenib (P = 0.070). For alopecia, lower risk was observed in comparisons against docetaxel (RR = 0.04, 95% CI: 0.01-0.11; P<0.001), while no significant difference was observed against trifluridine-tipiracil or regorafenib (P = 0.310). Compared with docetaxel, KRAS G12Ci was associated with a lower risk of all-grade asthenia (RR = 0.60, 95% CI: 0.36-0.98; P = 0.040). In comparisons against trifluridine-tipiracil or regorafenib, KRAS G12Ci was associated with a lower risk of all-grade fatigue (RR = 0.43, 95% CI: 0.19-0.94; P = 0.030). No significant differences were observed for all-grade asthenia (P = 0.100 for trifluridine-tipiracil or regorafenib), all-grade fatigue (P = 0.400 for docetaxel), or grade ≥3 fatigue (P = 0.130 for trifluridine-tipiracil or regorafenib).

### Quality assessment

The overall risk of bias across the included studies was low (**eFigures 1**, **2**). Based on the GRADE assessment of the evidence, the certainty was high for PFS and ORR, and moderate for OS, which was downgraded for imprecision (**eTable 5**).

### Publication bias and sensitivity analysis

We performed several sensitivity analyses to assess the robustness of our findings. Leave-one-out analyses confirmed that no single study drove the overall estimates. Sensitivity analyses restricted to NSCLC trials only (excluding the CRC trial) and to monotherapy studies only (excluding the combination therapy trial) yielded results consistent with the primary analysis. Given the limited number of included studies fewer than ten, formal publication bias assessment using funnel plots or Egger’s test was not performed, as these methods have low statistical power under such conditions.

## Discussion

This IPD meta-analysis of RCTs pooled the currently available data on KRAS G12Ci in solid tumors. Data from 949 patients across 3 RCTs suggested that KRAS G12Ci had advantages in PFS and ORR for solid tumors when compared with other standard or routine treatments, across almost all patient subgroups. However, KRAS G12Ci failed to demonstrate statistically significant OS advantage over the control group. Several factors may explain this apparent discrepancy. First, substantial treatment crossover may have occurred, as patients in the control arms who progressed on chemotherapy often received subsequent lines of therapy, including KRAS G12Ci in some cases, which could prolong OS in the control group and reduce the observed OS difference. Second, OS is heavily influenced by subsequent lines of therapy beyond the studied treatment period, and the availability of effective post-progression treatments may dilute any OS benefit derived from earlier PFS gains. Third, the follow-up duration in the included trials may have been insufficient to fully capture OS differences, particularly given that the survival curves tended to separate only after an extended period. Fourth, improvements in PFS and ORR do not always translate into OS prolongation, especially when disease control is followed by rapid progression or when salvage therapies are highly effective. Clinically, these findings suggest that KRAS G12Ci are effective in controlling tumor growth and delaying progression, but their impact on ultimate survival may be modified by post-progression events. Therefore, these agents may be more appropriately positioned as later-line therapy where the goal is disease control and symptom management rather than cure.

Notably, our exploratory subgroup analysis revealed that patients with PD-L1<50% appeared to derive greater benefit from KRAS G12Ci monotherapy. This finding, while hypothesis-generating, points to a potential immunological dimension of KRAS G12Ci that warrants further investigation. Preclinical evidence has demonstrated that KRAS G12Ci remodel the tumor microenvironment to enhance anti-tumor immunity (27). For instance, sotorasib has been shown to reduce tumor-infiltrating immunosuppressive cells while markedly increased the infiltration and activity of antigen-presenting cells and T cells (28). Specifically, sotorasib increased number of total and proliferating CD3+ T cells, as well as total CD8+ T cells, and also enhanced the infiltration of macrophages and dendritic cells (28). Meanwhile, by upregulating the expression of chemokines such as CXCL10 and CXCL11, sotorasib promoted recruitment and activation of T cells and dendritic cells, leading to the improved immunosurveillance within tumors (28). Similar immunomodulatory effects have been reported for adagrasib. In a KRAS G12C-mutant CT26 syngeneic mouse model, adagrasib decreased intratumoral myeloid-derived suppressor cells and enhanced the infiltration of M1-polarized macrophages, dendritic cells, CD4+, and CD8+T cells, accompanied by marked tumor regression (29). These immunomodulatory effects provide a mechanistic basis for our observation that patients with PD-L1<50% appear to derive greater benefit from KRAS G12C inhibitor monotherapy. Similar results were demonstrated in CodeBreaK 100 clinical trial (30) which suggested PD-L1 negativity was associated with a long-term clinical benefit of sotorasib monotherapy. Although low or negative PD-L1 expression does not necessarily indicate an ‘immune-active’ microenvironment, it may reflect a state with less pre-existing PD-L1-mediated immunosuppression characterized by fewer regulatory T cells (Tregs) and MDSCs (31, 32). Consequently, in tumors with low or negative PD-L1 expression, KRAS G12C inhibitor monotherapy may face fewer immune-related barriers, allowing its direct anti-tumor effects to dominate and resulting in better clinical outcomes.

In contrast, high PD-L1 tumors often harbor a more profoundly immunosuppressive microenvironment, characterized by increased Tregs and MDSCs, as well as more dysfunctional effector T cells whose function may not be readily restored by microenvironmental remodeling. This complex and redundant immunosuppressive environment is unlikely to be fully reversed by KRAS G12Ci alone (6). Beyond immune factors, differences in baseline tumor biology may also contribute. High PD-L1 expression has been associated with higher Ki-67 proliferation index and greater metastatic burden, features linked to more aggressive disease and poorer response to monotherapy (33). Furthermore, co-occurring genomic alterations, such as TP53 mutations, are more frequent in high PD-L1 tumors and are independently associated with poor prognosis, which may further limit the efficacy of KRAS G12Ci monotherapy (34). By reversing T-cell exhaustion and promoting their proliferation and tumor infiltration, PD-1/PD-L1 inhibitors can complement the antitumor immune effects of KRAS G12Ci (35). Concurrently, KRAS G12Ci induce immunogenic cell death, releasing neoantigens that prime new T cell activation (36, 37), thereby creating a more favorable immunological landscape for PD-1/PD-L1 inhibitors to act upon. Therefore, in tumors with high PD-L1 expression, combining KRAS G12Ci with PD-1/PD-L1 inhibitors may represent a rational therapeutic strategy. This rationale is supported by clinical data. Results from the KRYSTAL-7 Phase II study indicated that NSCLC patients with PD-L1 ≥50% achieved an ORR of 63% and a disease control rate (DCR) of 84% when treated with adagrasib plus pembrolizumab (38). Similarly, the LOXO-RAS-20001 trial demonstrated that olomorasib plus pembrolizumab achieved an ORR of 82% in the PD-L1 ≥50% subgroup (39). Therefore, we postulate that for KRAS G12Ci monotherapy, patients with low PD-L1 expression may be more likely to benefit, whereas in patients with high PD-L1 expression, combining KRAS G12Ci with PD-1 or PD-L1 inhibitors may be the path forward.

Beyond upfront combination, PD-1 or PD-L1 blockade may also help address acquired resistance to KRAS G12Ci, which remains a major clinical bottleneck (51). Mechanically, it is primarily driven by intratumoral heterogeneity, where a subpopulation of cells escapes treatment by synthesizing new KRAS G12C proteins that are maintained in their active GTP-bound state through signaling via EGFR or Aurora Kinase A (19, 40). Beyond this adaptive mechanism, resistance is further mediated by the activation of multiple receptor tyrosine kinases that converge on the downstream effector SHP2 to reactivate wild-type RAS signaling (41, 42). Additional pathways, including PI3K-AKT-mTOR activation, induction of epithelial-mesenchymal transition (EMT) (43), and dysregulation of the KEAP1-NRF2 antioxidant response pathway (44, 45), also contribute to a multifaceted resistance network. KRAS G12C-mutant tumors exhibit baseline immunosuppressive features, including upregulation of PD-L1 (6). Moreover, prolonged exposure to KRAS G12Ci can further induce adaptive immune escape mechanisms, characterized by additional PD-L1 upregulation, JAK2/STAT3/IL-6-mediated expansion of MDSCs, and reduced CD8+ T cell infiltration (22). These treatment-emergent changes establish a progressively immunosuppressive microenvironment that sustains acquired resistance. Encouragingly, sequential or concurrent administration of a PD-L1 inhibitor has been shown to reprogram this microenvironment, restore antitumor immunity, and re-sensitize resistant tumors to KRAS G12Ci (46). Although the current evidence for combining KRAS G12Ci with PD-L1 inhibitors is primarily derived from preclinical models, we propose that this combination strategy holds considerable promise for overcoming acquired resistance and merits further clinical investigation (**eTable 6**).

It is worth noting that in our study, while no significant OS benefit was observed in the overall population, KRAS G12Ci did demonstrate OS advantage in two specific subgroups: patients aged ≥65 years and those without liver metastases. Two types of accounts could be proposed for the OS benefit in liver metastases subgroup. The first hypothesis posits that liver metastases may induce specific mutations or activate alternative survival pathways that bypass KRAS inhibition. Research has shown that epithelial-mesenchymal transition can contribute to acquired resistance to KRAS G12Ci over time, which may not be as common in patients without liver metastases where the disease is less advanced or less resistant (47, 48). The second account concerns pharmacokinetics. KRAS G12Ci, like many targeted therapies, rely on effective drug delivery to the tumor site. In liver metastases, the blood supply and the tumor vasculature may not be as conducive to optimal drug penetration (4). Additionally, the liver’s metabolic processes may modify or clear drugs more rapidly, reducing their efficacy (49). Corroborating our findings, the phase 3 BREAKWATER study also demonstrated a significant OS improvement with targeted therapy in patients without liver metastases (HR = 0.36; 95% CI: 0.23-0.57) (50). In this context, we hypothesize that liver metastasis status may become a prognostic indicator for OS outcomes. As for ages, our study suggested patients aged ≥65 years with solid tumors experienced better OS benefits when treated with KRAS G12Ci, indicating that KRAS G12Ci may be more suitable for later-line therapy. This positioning is mirrored by the guidelines from the NCCN and the CSCO, which specify KRAS G12Ci as a second-line or later option following platinum-based chemotherapy in NSCLC (51). One speculative hypothesis is that immunosenescence, the age-related decline in immune function, may create an “immune-cold” tumor microenvironment that is more susceptible to KRAS G12C inhibition (52–54). However, we acknowledge that alternative explanations are equally plausible, including confounding by treatment line, differences in comorbidity burden, or the possibility that older patients may present with less aggressive disease biology at later lines. Due to limited number of studies included in subgroup analysis, these hypotheses may warrant further investigation in prospective studies.

It is encouraging that KRAS G12Ci exhibit a more favorable toxicity profile compared with standard treatments. Nevertheless, it’s important to note that KRAS G12Ci does give rise to diarrhoea that warrants attention. In our analysis, compared with docetaxel, KRAS G12Ci monotherapy was associated with a significantly higher risk of diarrhea (RR = 1.76). Gastrointestinal events, including diarrhea, vomiting, and nausea, represented the most commonly reported AEs with adagrasib, as confirmed by previous research (50). However, these were largely Grade 1 and could be resolved rapidly with appropriate strategies, such as dose modifications, topical or systemic therapies, and close monitoring. Notably, when compared with trifluridine-tipiracil or regorafenib which also carry significant gastrointestinal toxicity, the risk of diarrhea was comparable (RR = 0.96), suggesting that the gastrointestinal burden of KRAS G12Ci is similar to that of other standard-of-care later-line therapies for CRC. Rash also deserves consideration (RR = 14.43). Nevertheless, rash is generally manageable with prophylactic skin care and supportive therapies. Notably, the elevated risk of rash may be partly attributable to the concomitant use of panitumumab, an epidermal growth factor receptor (EGFR) inhibitor for which rash is the most common adverse event (55). Overall, KRAS G12Ci has a relatively mild side effect profile, which offers a wider therapeutic window to incorporate additional agents. This, in turn, helps reduce the overall burden of treatment-related toxicity, making KRAS G12Ci an attractive candidate for combination regimens.

Despite the low statistical heterogeneity observed for both OS and PFS (I²=0%), the clinical heterogeneity arising from the inclusion of two distinct tumor types and varied treatment regimens warrants careful consideration. Although NSCLC and CRC are biologically distinct malignancies, they share KRAS G12C as a common oncogenic driver. The mechanism of KRAS G12Ci is target-specific rather than tumor type-specific, acting by locking the protein in its inactive GDP-bound state. Preclinical evidence has demonstrated that KRAS G12Ci exert on-target effects across various KRAS G12C-mutant malignancies, with antitumor activity primarily determined by the presence of the G12C mutation rather than tissue of origin. Thus, pooling these two tumor types allows for evaluation of the class effect of KRAS G12Ci across the broader KRAS G12C-mutant population, an approach aligned with the emerging paradigm of tissue-agnostic drug development. We acknowledge, however, the biological differences between NSCLC and CRC, as well as the imbalance in sample size that weights the pooled estimates toward NSCLC. Subgroup analysis by tumor type showed no statistically significant interaction (P = 0.444), and the PFS HR for the CRC cohort (HR = 0.53, 95% CI 0.38-0.75) was directionally consistent with that of the NSCLC cohort (HR = 0.62, 95% CI 0.51-0.74), with overlapping confidence intervals, suggesting consistent treatment effects across tumor types. A further layer of complexity arises from differences in treatment regimens: CRC patients received KRAS G12Ci in combination with the EGFR inhibitor panitumumab, whereas NSCLC patients received KRAS G12Ci as monotherapy. This introduces potential clinical and biological confounding that may influence the observed outcomes in the CRC cohort. EGFR inhibition may enhance antitumor efficacy or modify safety profiles, and the limited number of eligible trials prevents full stratification by combination versus monotherapy. To address this, sensitivity analyses excluding the CRC studies were performed, yielding a PFS HR of 0.62 (95% CI 0.51-0.74) for NSCLC monotherapy alone, which remained statistically significant and directionally consistent with the main pooled estimate (HR = 0.60, 95% CI 0.51-0.70). Although the CRC cohort showed a numerically lower HR (0.53, 95% CI 0.38-0.75) compared with the NSCLC cohort (0.62, 95% CI 0.51-0.74), the 95% confidence intervals overlapped substantially, and the interaction test was not significant (P = 0.444). These analyses suggest that while combination therapy may enhance efficacy in CRC, the overall pooled estimate remains a reasonable and conservative reflection of the benefit conferred by KRAS G12C inhibition across both monotherapy and combination therapy settings. Collectively, these findings indicate that despite inherent clinical heterogeneity related to tumor type, treatment regimen, and underlying disease biology, the overall conclusions of this meta-analysis are robust. Nevertheless, the class-wide KRAS G12Ci effects should be interpreted with caution, as combination therapy and tumor-specific biological factors may still contribute to residual confounding.

## Strengths and limitations

This meta-analysis has some strengths. The primary advantage is the utilization of IPD, which standardize how outcomes are measured across studies, addressing study-level heterogeneity. In addition, the meta-analysis of individual participant time-to-event data yielded more precise and reliable pooled estimates than conventional meta-analyses based on aggregate data. Furthermore, the validity of the findings is reinforced by their consistency across two-stage meta-analyses. Finally, the robustness of the results is underscored by the negligible between-study heterogeneity (I²=0.0% for both PFS and OS) and the high-quality evidence rating according to the GRADE framework.

Several limitations of this study should also be acknowledged. First, as the IPD were reconstructed from published K-M curves, we were unable to perform subgroup analyses using original baseline clinical variables. Future IPD meta-analyses incorporating patient-level data obtained directly from study authors are warranted. Second, the limited number of eligible studies precluded additional subgroup analyses, including by treatment timing, and prevented meaningful subgroup analysis for ORR. Thirdly, pooling liver metastasis subgroup estimates across different tumor types introduces clinical heterogeneity. Moreover, the small number of studies in each subgroup inevitably limited statistical power. Furthermore, multiple subgroup analyses were conducted without adjustment for multiple comparisons, which increases the risk of type I error. Therefore, these subgroup findings should be interpreted as exploratory and hypothesis-generating rather than confirmatory. Additionally, the inclusion of NSCLC and CRC trials, with differences in tumor biology and treatment regimens (monotherapy versus EGFR combination), introduces potential clinical and biological confounding that cannot be fully addressed statistically. These methodological constraints underscore the need for future multi-center randomized trials with extended follow-up and careful monitoring of attrition to strengthen the evidence base.

## Conclusion

KRAS G12Ci exhibit antitumor activity in solid tumors and a generally favorable safety profile compared with non-KRAS G12Ci treatments, with diarrhea and rash remaining notable adverse events. While no significant OS benefit was observed in the overall population, improvements in PFS and ORR indicate potential utility in later-line settings. Subgroup analyses suggest that patients aged ≥65 years, those without liver metastases, or those with PD-L1 expression <50% may represent populations for enrichment in future clinical trials. These observations are hypothesis-generating and may inform patient enrichment strategies in future clinical trials, helping to guide the selection of populations for more precise evaluation of KRAS G12Ci efficacy.

99 Lu F, Wang E, Liu H. Factors correlating the expression of PD-L1. BMC Cancer. 2024 May 25;24 (1):642. doi: 10.1186/s12885-024-12400-9. PMID: 38796458; PMCID: PMC11127358.

100 Dong ZY, Zhong WZ, Zhang XC, Su J, Xie Z, Liu SY, Tu HY, Chen HJ, Sun YL, Zhou Q, Yang JJ, Yang XN, Lin JX, Yan HH, Zhai HR, Yan LX, Liao RQ, Wu SP, Wu YL. Potential Predictive Value of TP53 and KRASMutation Status for Response to PD-1 Blockade Immunotherapy in Lung Adenocarcinoma. Clin Cancer Res. 2017 Jun 15;23 (12):3012-3024. doi: 10.1158/1078-0432.CCR-16-2554. Epub 2016 Dec 30. PMID: 28039262.

## Data Availability

The raw data supporting the conclusions of this article will be made available by the authors, without undue reservation.
