# Supplementary material for: KRAS G12C inhibitors in KRASG12C-mutated solid tumors: an immunologically informed systematic review and reconstructed individual patient data meta-analysis
Source: Front Immunol. 2026 Jul 10;17:1848431. doi: 10.3389/fimmu.2026.1848431 (PMC13396023; doi:10.3389/fimmu.2026.1848431)
Supplement: Supplementary file 1 [file DataSheet1.docx]

**Supplementary Online Content**

KRAS G12C Inhibitors in KRAS^G12C^-Mutated Solid Tumors: A Systematic Review and Reconstructed Individual Patient Data Meta-Analysis

**eTable 1.** Detailed Search Strategy

**eTable 2.** Interaction P value of subgroup analysis

**eTable 3.** Characteristics of included articles

**eTable 4.** Pooled safety analyses across all studies in this meta-analysis

**eTable 5.** Quality assessment of the evidence of the included publications, based on the GRADE

**eTable 6.** Overview of Resistance Mechanisms to KRAS G12Ci

**eFigure 1.** Risk of Bias Graph: Reviews of Authors’ Judgments about Each Risk of Bias Item Are Presented as Percentages Across all Included Studies

**eFigure 2.** Risk of Bias Summary: Reviews of Authors’ Judgments about Each Risk of Bias Item for Each Included Study

**eFigure 3.** Funnel Plot of PFS

**eFigure 4.** Funnel Plot of OS

**eFigure 5.** Funnel Plot of ORR

**eAppendix 1.** Reconstructed Survival Curve and Side-by-Side Comparison with the Original Curve, as well as Difference Between Estimated and Read-In Survival Probabilities for Each Included Study

This supplementary material has been provided by the authors to give readers additional information about their work.

**eTable 1.** Detailed Search Strategy

**Data searched from PubMed**

| **Search Query** | **Results** |
| --- | --- |
| #1 "Neoplasms"[Mesh] OR neoplas* OR tumor* OR cancer* OR malignan* OR tumour* OR carcinom* OR adenocarcin* | 6,225,983 |
| #2 "KRAS G12C Inhibitor" OR Sotorasib OR LUMAKRAS OR LUMYKRAS OR "AMG 510" OR Adagrasib OR Krazati OR MRTX849 OR Fulzerasib OR GFH925 OR IBI351 OR Garsorasib OR D-1553 OR Glecirasib OR JAB-21822 | 860 |
| #3#1 AND #2 | 765 |

Searched before March 28, 2026. Results: 725.

**Data searched from** **Embase**

| **Search Query** | **Results** |
| --- | --- |
| #1 'neoplasm'/exp OR neoplas* OR tumor* OR cancer* OR malignan* OR tumour* OR carcinom* OR adenocarcin* | 9,121,434 |
| #2 'KRAS G12C Inhibitor' OR Sotorasib OR LUMAKRAS OR LUMYKRAS OR 'AMG 510' OR Adagrasib OR Krazati OR MRTX849 OR Fulzerasib OR GFH925 OR IBI351 OR Garsorasib OR D-1553 OR Glecirasib OR JAB-21822 | 2,543 |
| #3 #1 AND #2 | 2,463 |

Searched before March 28, 2026. Results: 2,353.

**Data searched from Cochrane Library**

| **Search Query** | **Results** |
| --- | --- |
| #1 MeSH descriptor: [Neoplasms] explode all trees | 130,409 |
| #2(cancer):ti,ab,kw OR (tumor):ti,ab,kw OR (adenocarcinoma):ti,ab,kw | 255,796 |
| #3 #1 OR #2 | 286,914 |
| #4 ("KRAS G12C Inhibitor"):ti,ab,kw OR (Sotorasib):ti,ab,kw OR (LUMAKRAS):ti,ab,kw OR (LUMYKRAS):ti,ab,kw OR ("AMG 510"):ti,ab,kw OR (Adagrasib):ti,ab,kw OR (Krazati):ti,ab,kw OR (MRTX849):ti,ab,kw OR (Fulzerasib):ti,ab,kw OR (GFH925):ti,ab,kw OR (IBI351):ti,ab,kw OR (Garsorasib):ti,ab,kw OR (D-1553):ti,ab,kw OR (Glecirasib):ti,ab,kw OR (JAB-21822):ti,ab,kw | 121 |
| #5 #3 OR #4 | 101 |

Searched before March 28, 2026. Results: 96.

**eTable 2.** Interaction P value of subgroup analysis

|  | Subgroup | Interaction P value |
| --- | --- | --- |
| PFS | Age | 0.690 |
|  | Body site | 0.889 |
|  | Bone metastasis | 0.819 |
|  | ECOG score | 0.513 |
|  | Histology | 0.444 |
|  | Previous line | 0.576 |
|  | Liver metastasis | 0.030 |
|  | Location of tumor | 0.583 |
|  | PD-L1 expression | 0.730 |
|  | Race | 0.569 |
|  | Regimen type | 0.444 |
|  | Sex | 0.759 |
| OS | Age | 0.005 |
|  | Body site | 0.746 |
|  | Previous line | 0.983 |
|  | Liver metastasis | 0.158 |
|  | Location of tumor | 0.711 |
|  | Sex | 0.439 |

PFS, progression-free survival; OS, overall survival; ECOG, Eastern Cooperative Oncology Group; PD-L1, Programmed Death Ligand 1.

**eTable 3.** Characteristics of included articles

| **Source** | **Design** | **Masking** | **Phase** | **Stage** | **Median Follow-up, months** | **No. of**  **Patients** | **Regimen** | **No. of**  **male (%)** | **Median age, years** | **ORR** |
| --- | --- | --- | --- | --- | --- | --- | --- | --- | --- | --- |
| Langen, 2023 | multicenter | open-label | 3 | Metastatic/ Advanced | 17.7 | 345 | I: Sotorasib | 109 (63.7%) | 64.0 | 28.1% (21.5-35.4) |
|  |  |  |  |  |  |  | C: Docetaxel | 95 (54.5%) | 64.0 | 13.2% (8.6-19.2) |
| Fakih, 2023 | multicenter | open-label | 3 | Metastatic | 7.8 | 160 | Ia:Sotorasib+Panitumumab | 29 (54.7%) | 63.0 | 26.4% (15.3-40.3) |
|  |  |  |  |  |  |  | Ib: Sotorasib+Panitumumab | 26 (49.1%) | 58.0 | 5.7% (1.2-15.7) |
|  |  |  |  |  |  |  | C: Trifluridine-tipiracil/ Regorafenib | 24 (44.4%) | 64.5 | 0.0% (0.0-6.6) |
| Pietrantonio, 2025 | multicenter | open-label | 3 | Metastatic | 13.6 | 160 | Ia:Sotorasib+Panitumumab | 29 (54.7%) | 63.0 | 30.2% (18.3-44.3) |
|  |  |  |  |  |  |  | Ib: Sotorasib+Panitumumab | 26 (49.1%) | 58.0 | 7.5% (2.1-18.2) |
|  |  |  |  |  |  |  | C: Trifluridine-tipiracil/ Regorafenib | 24 (44.4%) | 65.0 | 1.9% (0.0-9.9) |
| Barlesi, 2015 | multicenter | open-label | 3 | Metastatic/ Advanced | 7.2 | 453 | I: Adagrasib | 193 (64.1%) | 64.0 | 32.0% (26.7-37.5) |
|  |  |  |  |  |  |  | C: Docetaxel | 110 (72.3%) | 65.0 | 9.0% (5.1-15.0) |

I, intervention group; C, control group; No, numbers; ORR, objective Response Rate.

**eTable 4**. Pooled safety analyses across all studies in this meta-analysis

| Adverse events | | RR (95% CI) | | | |
| --- | --- | --- | --- | --- | --- |
|  |  | All Grade | P value | Grade≥3 | P value |
| Digestive system | Constipation | 0.68 (0.19, 2.40) | 0.550 | NA | NA |
|  | Decreased appetite | 0.93 (0.69, 1.25) | 0.620 | NA | NA |
|  | Diarrhoea | 1.52 (1.15, 2.02) | 0.003 | 1.60 (0.69, 3.70) | 0.280 |
|  | Nausea | 0.80 (0.42, 1.56) | 0.520 | 2.29 (0.59, 8.83) | 0.230 |
|  | Vomiting | 1.40 (0.43, 4.59) | 0.580 | NA | NA |
| Hematological system | Anaemia | 0.44 (0.18, 1.08) | 0.070 | 0.60 (0.27, 1.32) | 0.200 |
|  | Leukopenia | 0.09 (0.02, 0.46) | 0.040 | 0.19 (0.02, 1.62) | 0.130 |
|  | Neutropenia | 0.12 (0.04, 0.32) | <0.001 | 0.12 (0.05, 0.31) | <0.001 |
|  | Thrombocytopenia | 0.37 (0.11, 1.28) | 0.120 | NA | NA |
| Skin | Alopecia | 0.10 (0.02, 0.42) | 0.002 | NA | NA |
|  | Rash | 4.55 (0.50, 41.22) | 0.180 | 6.74 (0.84, 53.84) | 0.070 |
| Others | Asthenia | 0.53 (0.36, 0.78) | 0.001 | NA | NA |
|  | Fatigue | 0.49 (0.22, 1.13) | 0.090 | 0.67 (0.15, 3.04) | 0.600 |

NA, not available.

**eTable 5.** Quality assessment of the evidence of the included publications, based on the GRADE

| **Quality assessment** | | | | | | | **No of patients** | | **Effect** | | **Quality** | **Importance** |  |
| --- | --- | --- | --- | --- | --- | --- | --- | --- | --- | --- | --- | --- | --- |
|  |  |  |  |  |  |  |  |  |  |  |  |  |  |
| **No of studies** | **Design** | **Risk of bias** | **Inconsistency** | **Indirectness** | **Imprecision** | **Other considerations** | **Intervention** | **Control** | **Relative (95% CI)** | **Absolute** |  |  |  |
| **PFS** | | | | | | | | | | | | |  |
| 4 | RCT | not serious | not serious | not serious | not serious | none | 578 | 434 | HR:0.60  (0.51,0.70) | 161/578 | ÅÅÅÅ HIGH | IMPORTANT |  |
|  |  |  |  |  |  |  |  |  |  |  |  |  |  |
| **OS** | | | | | | | | | | | | |  |
| 3 | RCT | not serious | not serious | not serious | serious | none | 277 | 282 | HR:0.92 (0.73,1.14) | 68/277 | ÅÅÅO  MODERATE | IMPORTANT |  |
| **ORR** | | | | | | | | | | | | |  |
| 4 | RCT | not serious | not serious | not serious | not serious | none | 684 | 542 | RR:3.60 (2.01,6.46) | 181/684 | ÅÅÅÅ   HIGH | IMPORTANT |  |

No, numbers; CI, confidence interval; PFS, progress-free survival; RCT, randomized controlled trial; HR, hazard ratio; RR, relative risk.

**eTable 6.** Overview of Resistance Mechanisms to KRAS G12Ci

| Category | Mechanism | Key Mediators / Pathways | Therapeutic Implication |
| --- | --- | --- | --- |
| Cell‑intrinsic adaptive mechanisms | Reactivation of KRAS signaling | New KRAS G12C protein synthesis via EGFR or Aurora Kinase A signaling | Combination with EGFR or Aurora Kinase A inhibitors |
|  | Bypass pathway activation | RTKs (EGFR, MET) converging on SHP2 to reactivate wild‑type RAS | Combination with SHP2 inhibitors |
|  | PI3K‑AKT‑mTOR activation | Upstream or downstream activation of the PI3K pathway | Combination with PI3K or mTOR inhibitors |
|  | EMT | Loss of epithelial markers, gain of mesenchymal phenotype | Combination with EMT‑targeting agents or chemotherapy |
|  | KEAP1‑NRF2 pathway dysregulation | Oxidative stress response activation | Potential synergy with antioxidant‑targeting therapies |
| Immune‑mediated mechanisms | Baseline immune suppression | PD‑L1 upregulation in KRAS G12C‑mutant tumors | PD‑1/PD‑L1 checkpoint blockade |
|  | Adaptive immune escape after prolonged KRAS G12Ci exposure | Additional PD‑L1 upregulation, JAK2/STAT3/IL‑6 mediated MDSC expansion, reduced CD8⁺ T cell infiltration | Sequential or concurrent PD‑L1 inhibitor therapy |
|  | Immunosuppressive microenvironment | Accumulation of MDSCs and Tregs, T cell exhaustion | Combination with MDSC‑targeting or Treg‑depleting agents |

EGFR, epidermal growth factor receptor; RTKs, receptor tyrosine kinases; SHP2, Src homology region 2 domain-containing phosphatase 2; PI3K, phosphatidylinositol 3-kinase; mTOR, mammalian target of rapamycin; EMT, epithelial‑mesenchymal transition; KEAP1, kelch‑like ECH‑associated protein 1; NRF2, nuclear factor erythroid 2‑related factor 2; PD‑L1, programmed death ligand 1; MDSCs, myeloid‑derived suppressor cells; JAK2, Janus kinase 2; STAT3, signal transducer and activator of transcription 3; IL‑6, interleukin 6; Tregs, regulatory T cells.

**eFigure 1.** Risk of Bias Graph: Reviews of Authors' Judgments About Each Risk of Bias Item Are Presented as Percentages Across all Included Studies


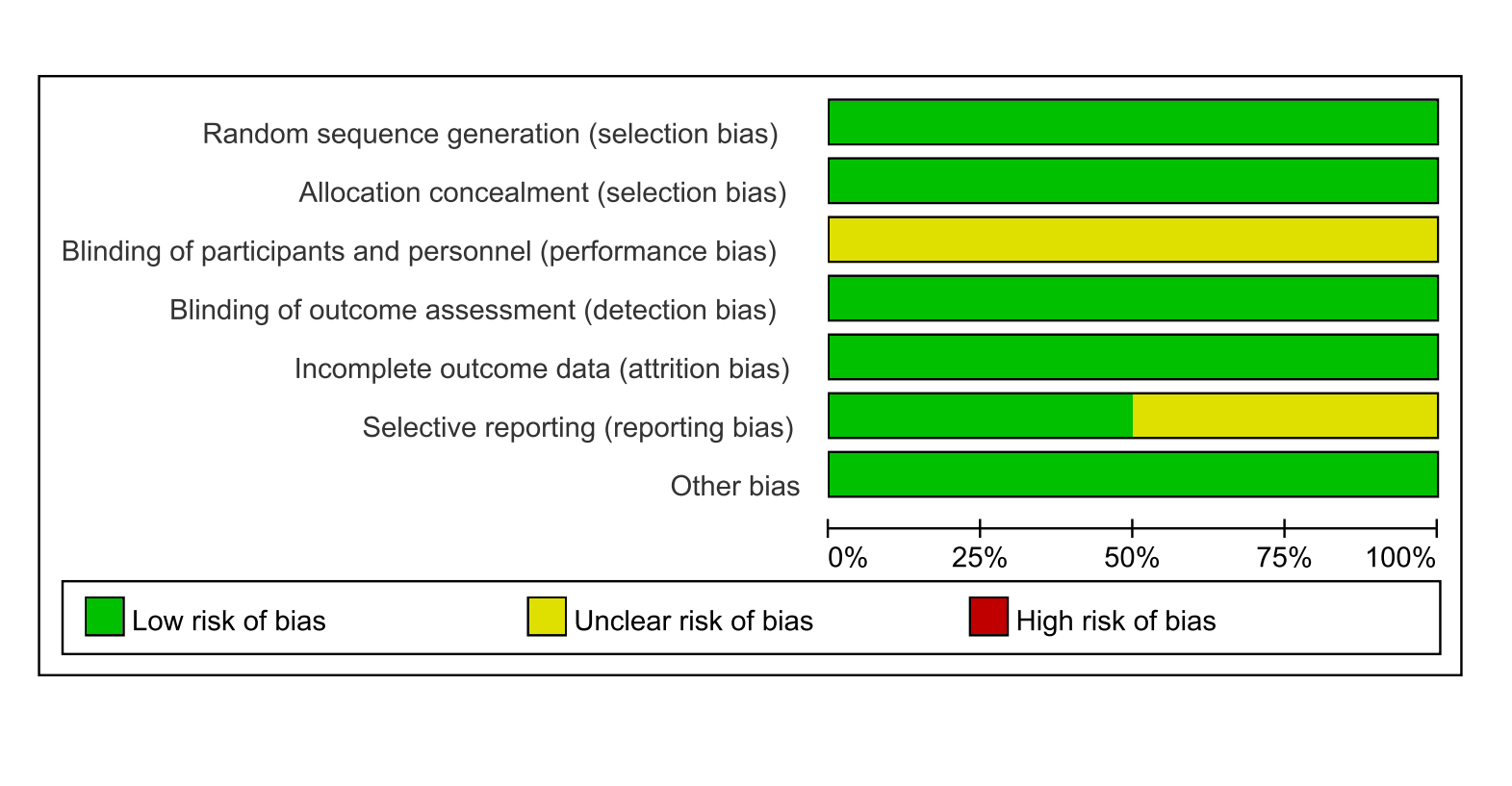


**eFigure 2.** Risk of Bias Summary: Reviews of Authors' Judgments About Each Risk of Bias Item for Each Included Study


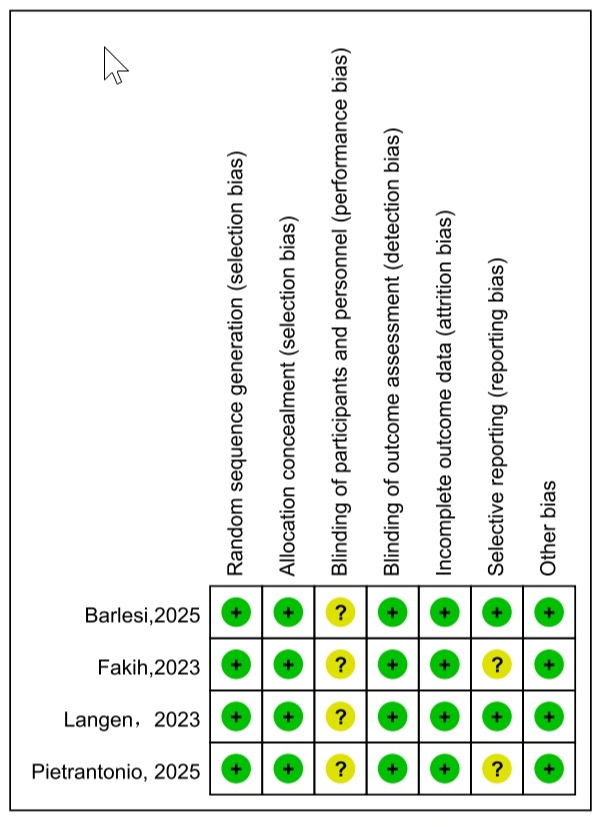


**eFigure 3.** Funnel Plot of PFS


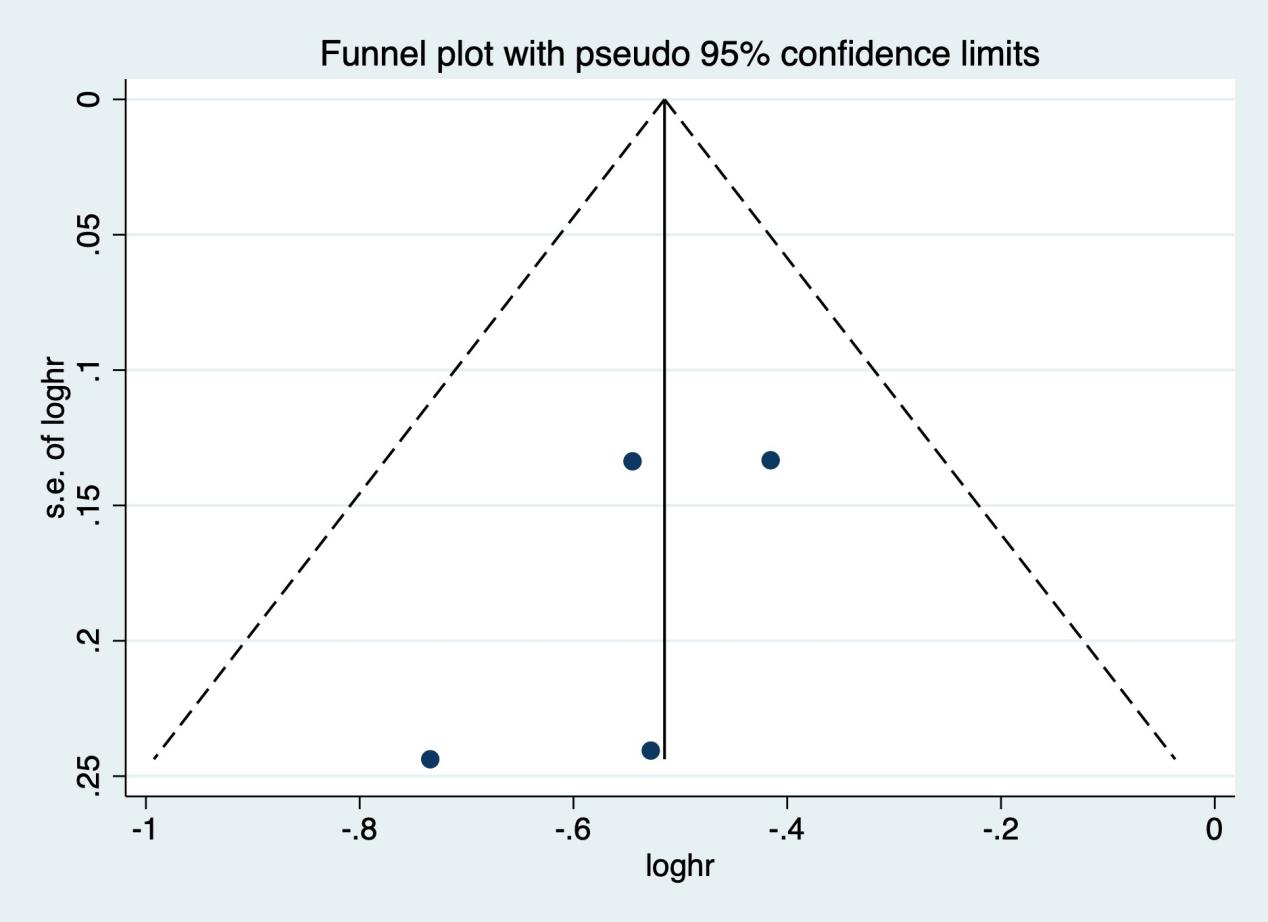


**eFigure 4.** Funnel Plot of OS


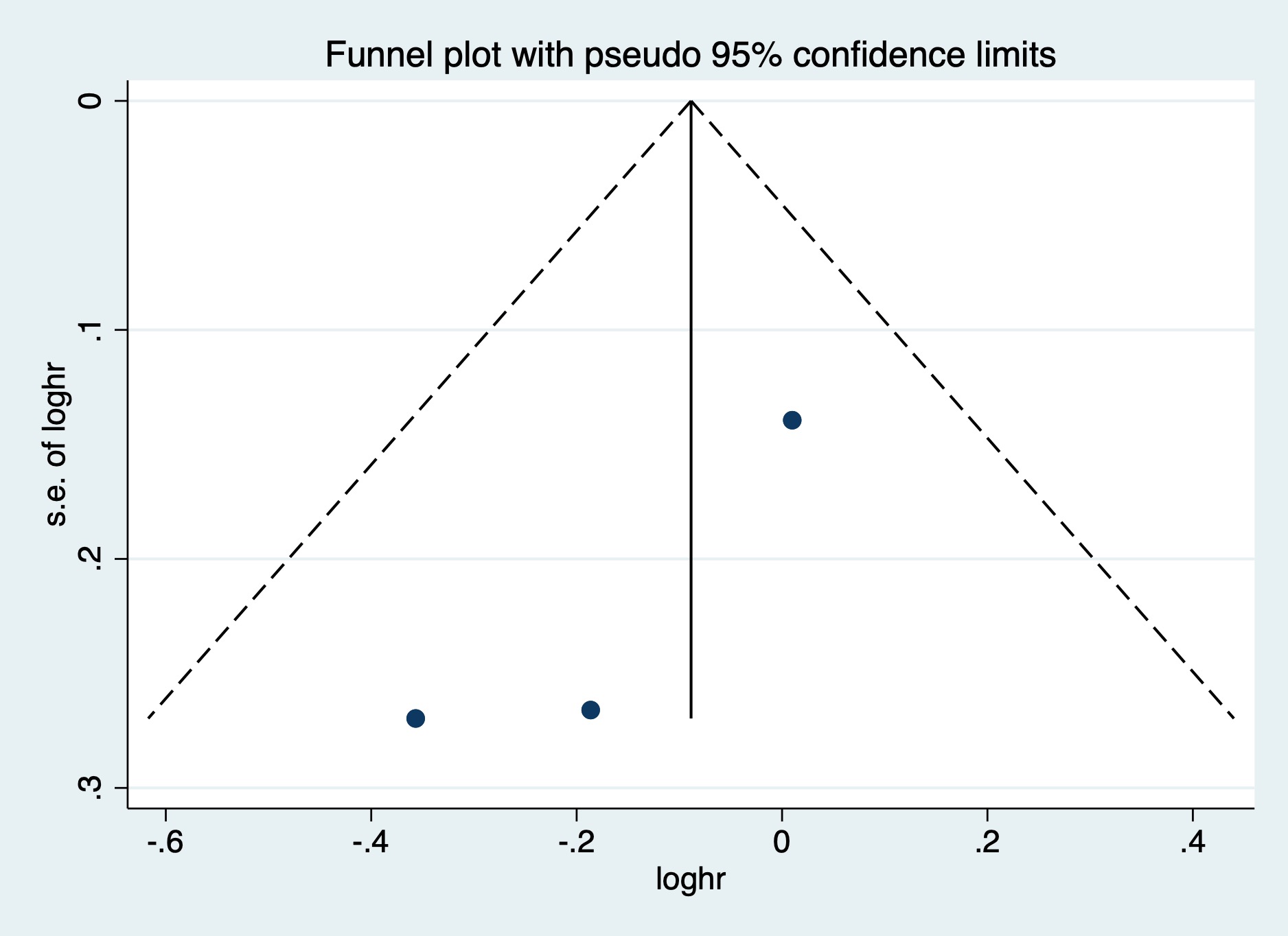


**eFigure 5.** Funnel Plot of ORR


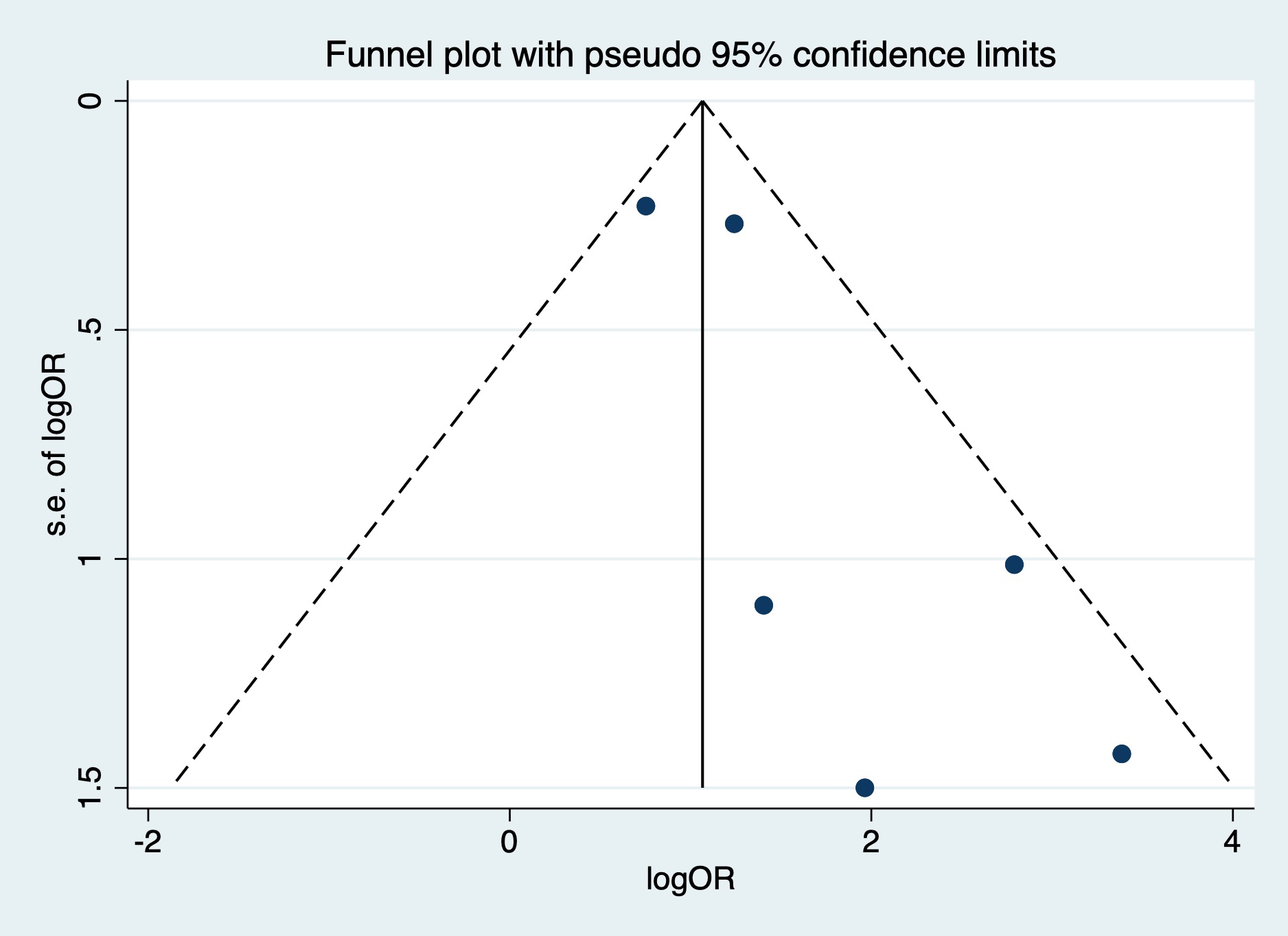


**eAppendix 1.** Reconstructed Survival Curve and Side-by-Side Comparison with the Original Curve as well as Difference Between Estimated and Read-In Survival Probabilities for Each Included Study*

*The curve above is the reconstructed curve, and the curve below is the original published curve.

Abbreviations: PFS, progress-free survivall; OS, overall survival.

**Barlesi, 2025-PFS**


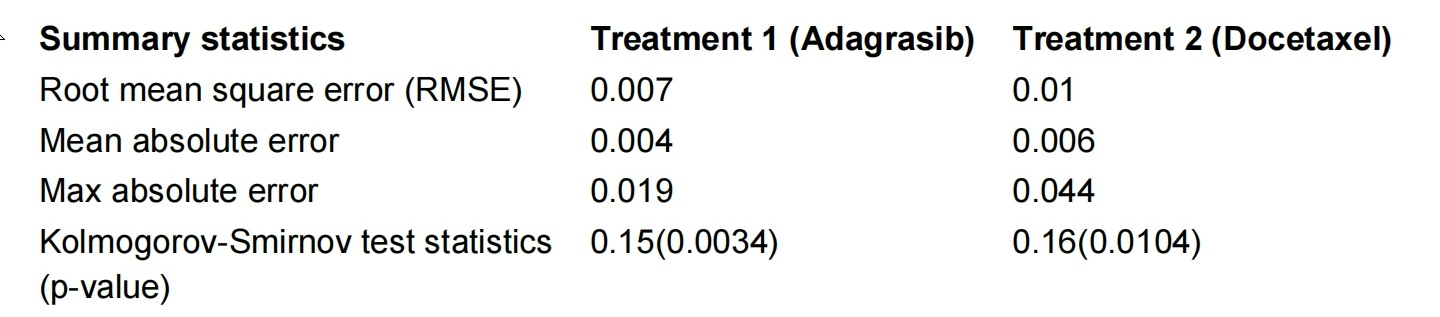


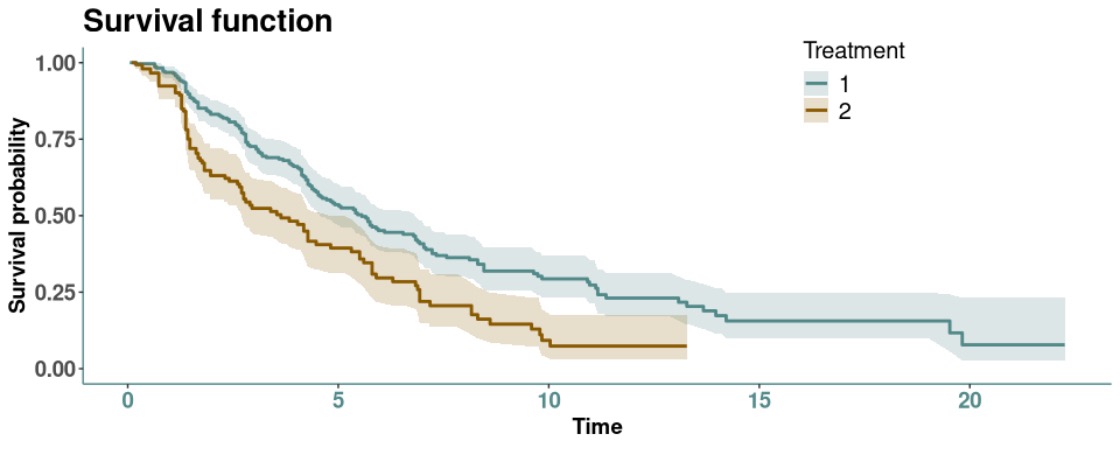


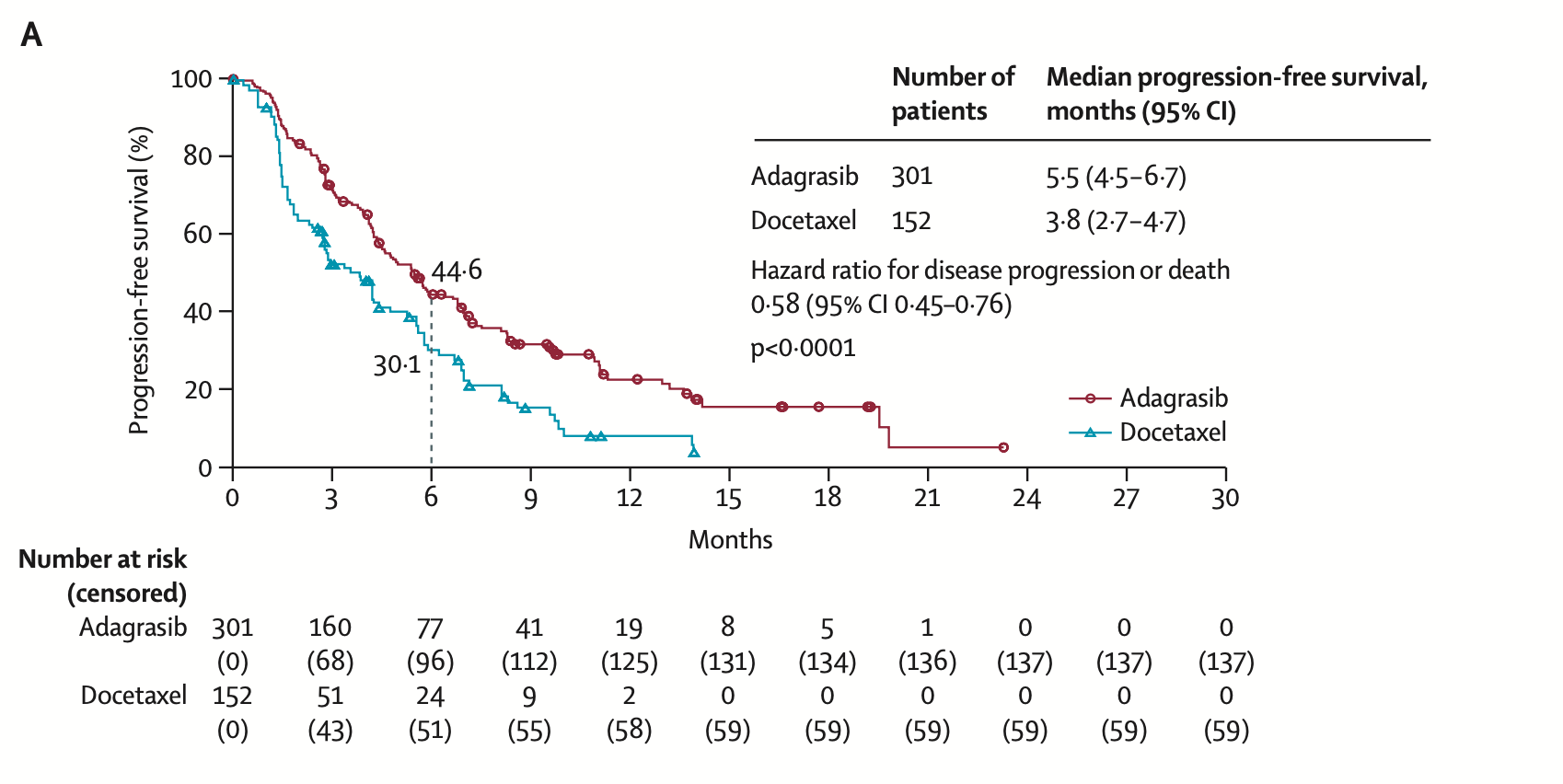


**Langen, 2023-OS**


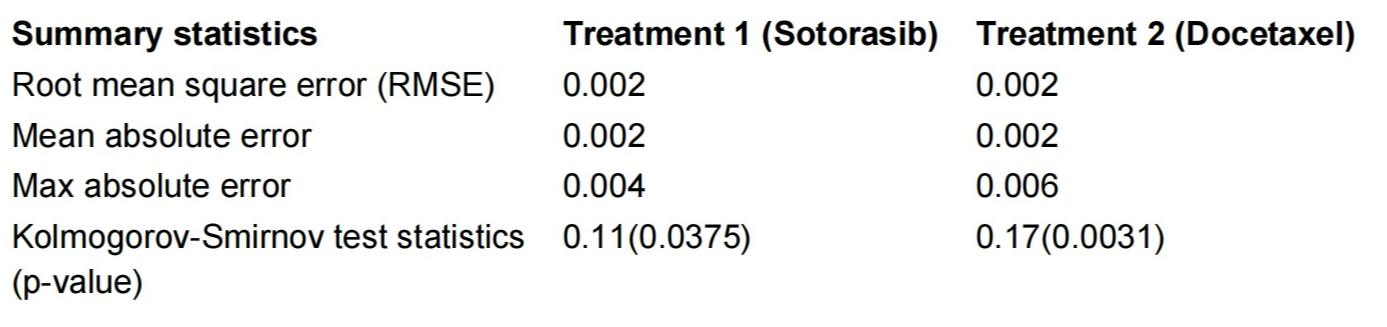


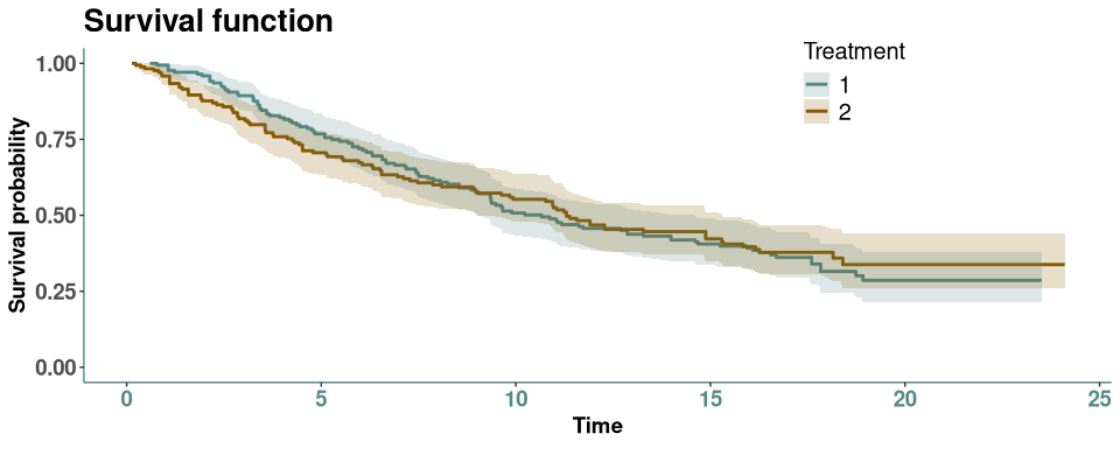


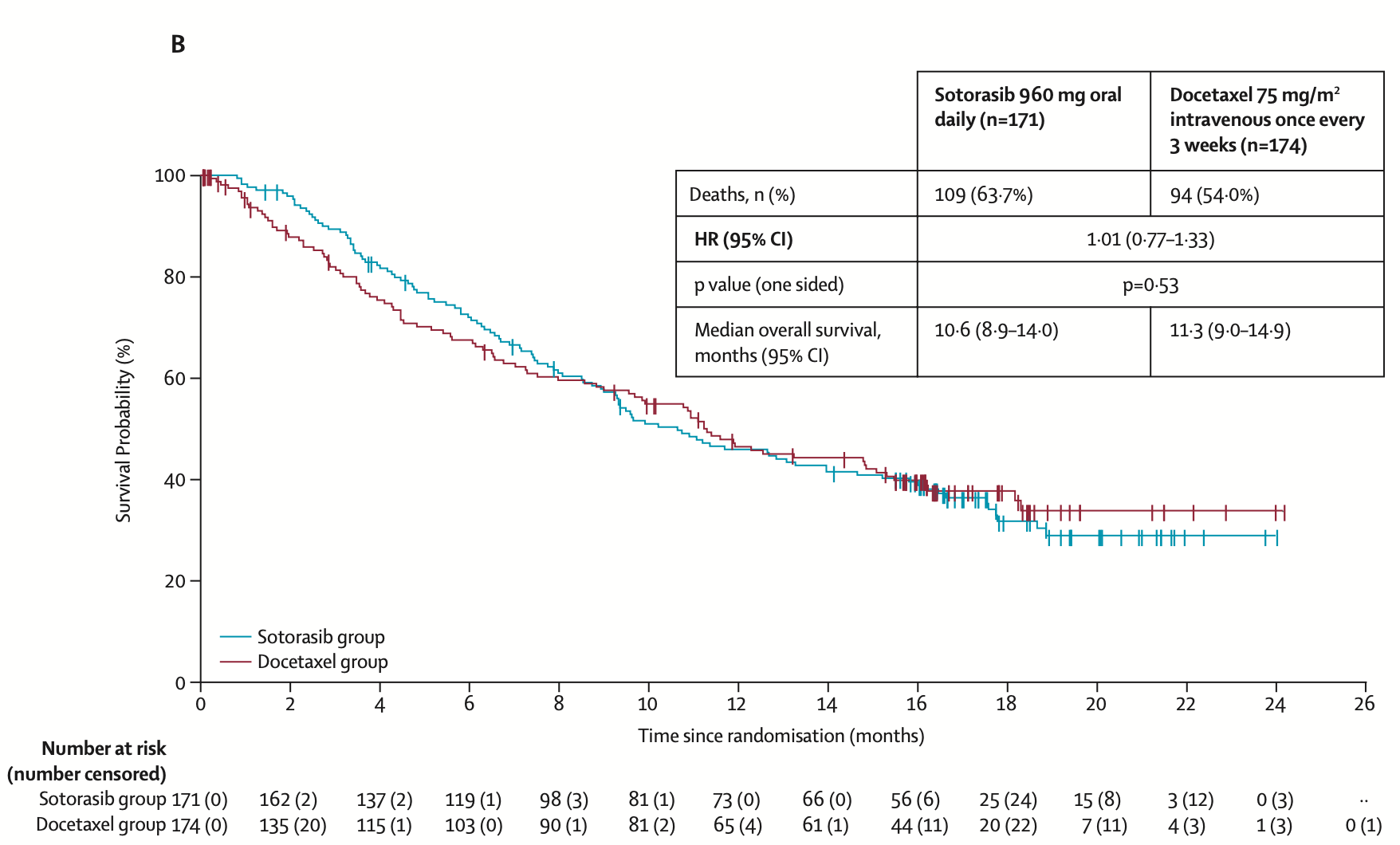


**Langen, 2023-PFS**


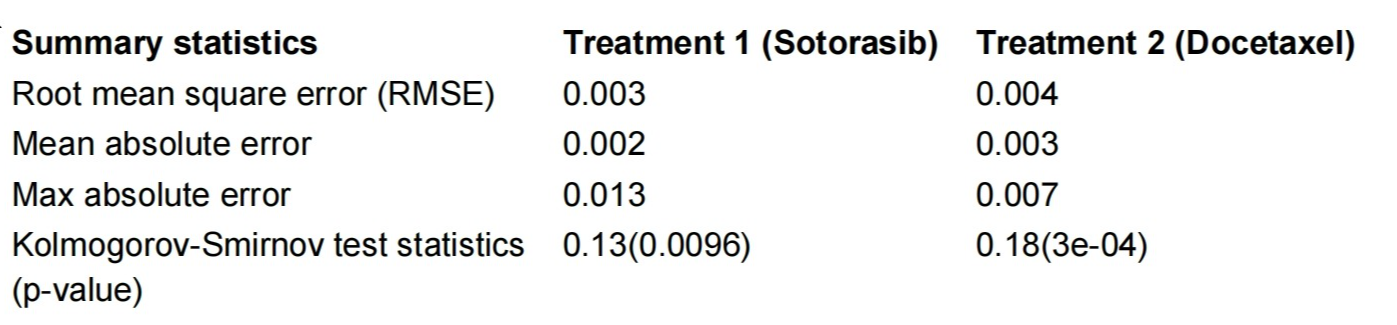


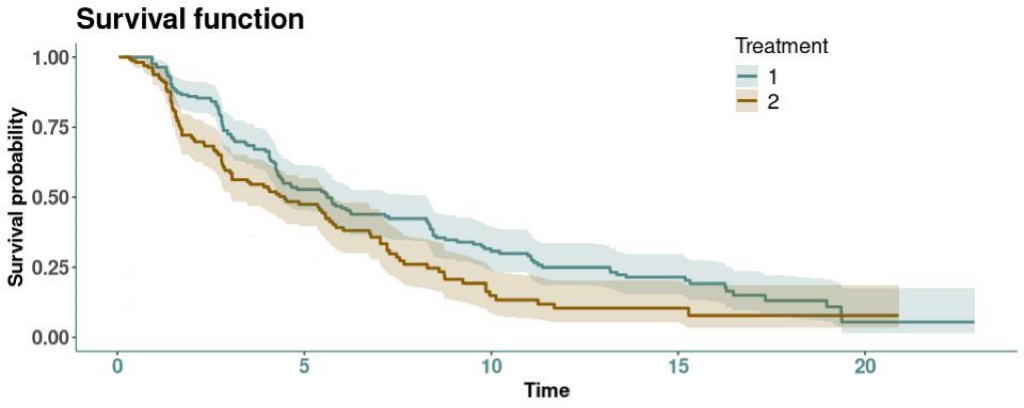


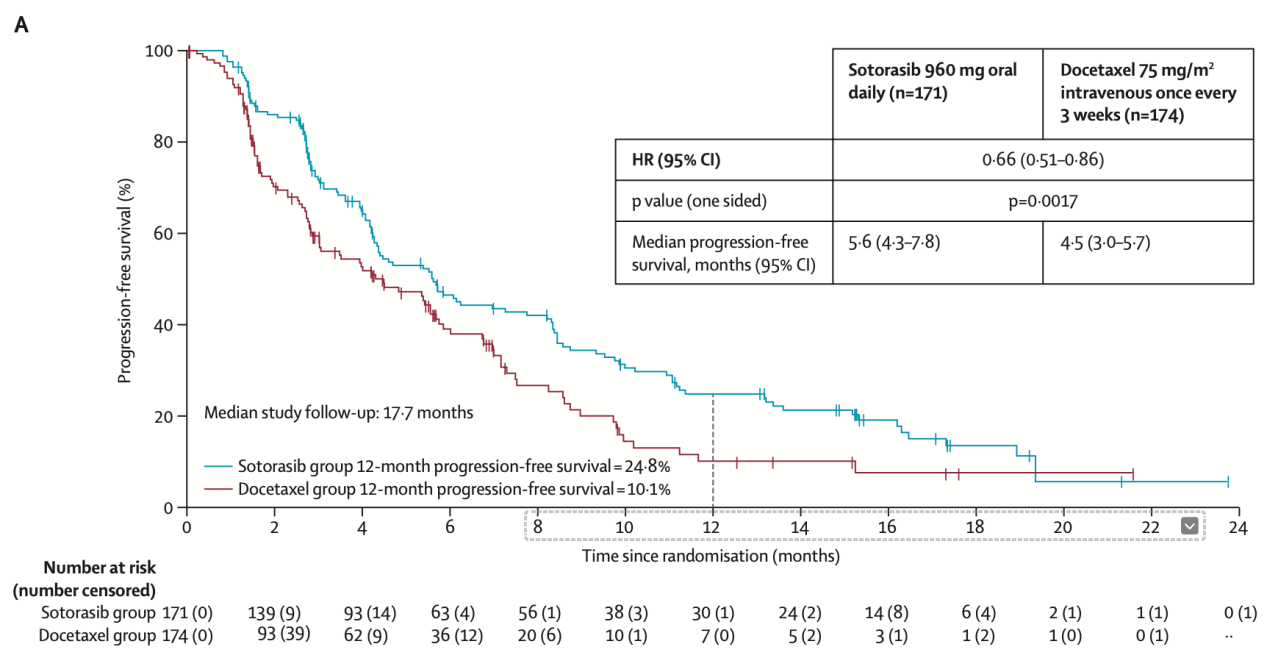


**Pietrantonio, 2025-OS (Sotorasib 240mg)**

**
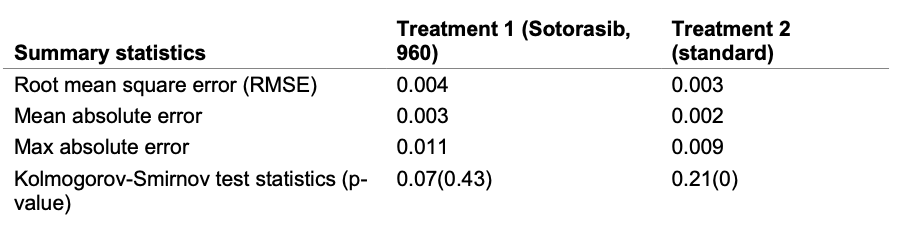
**


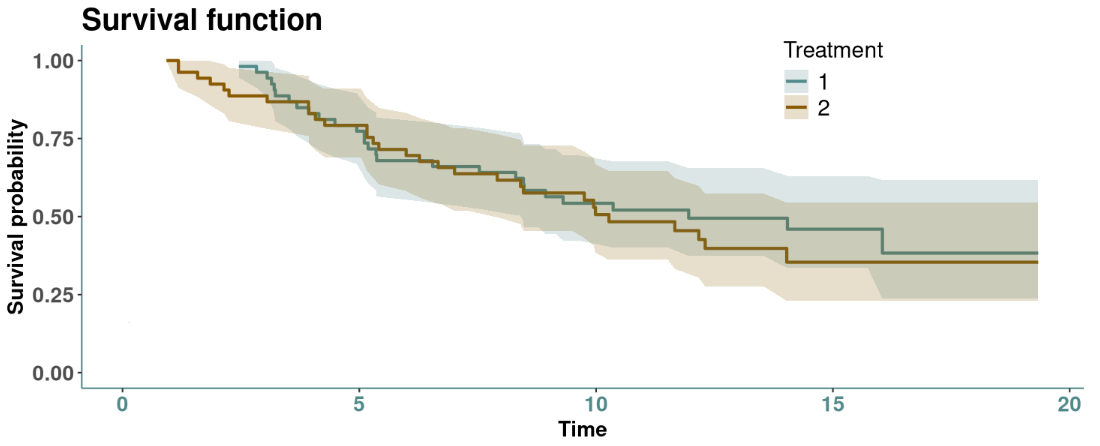


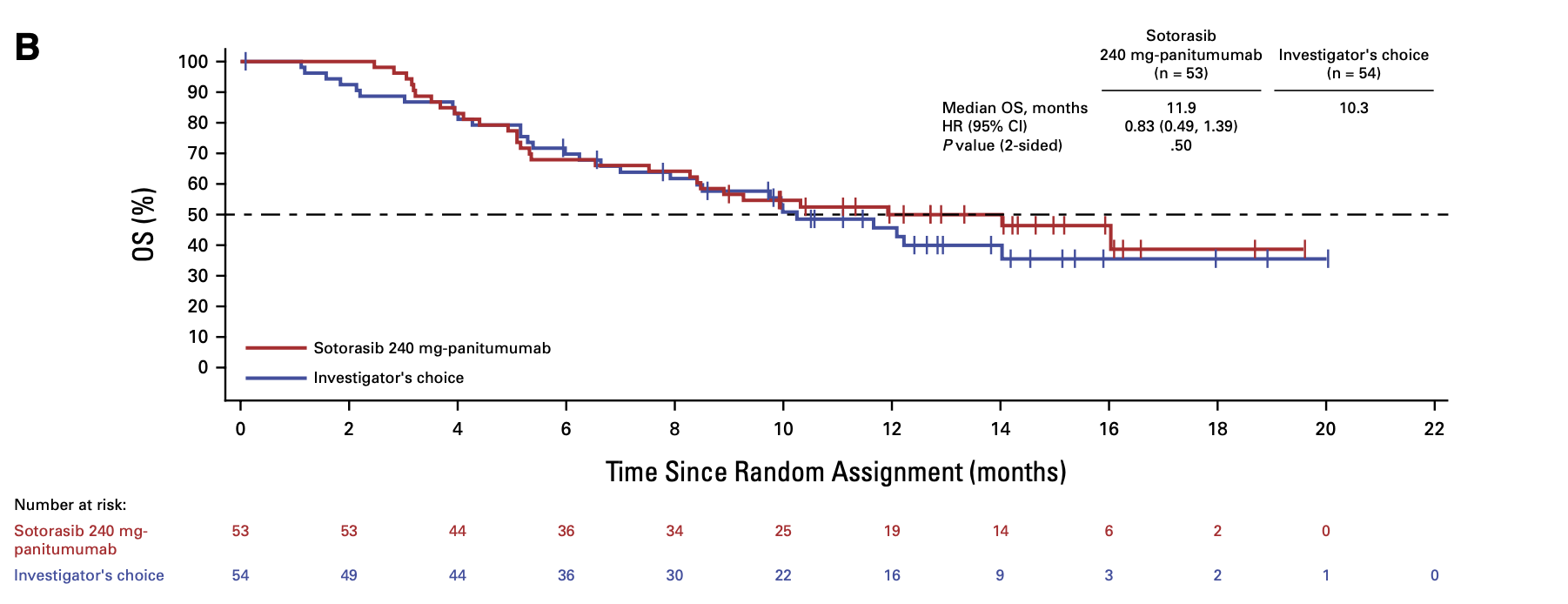


**Pietrantonio, 2025-OS (Sotorasib 960mg)**


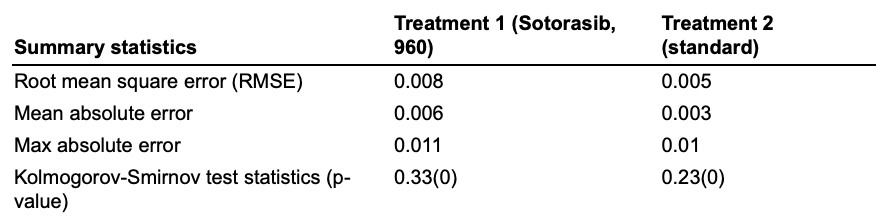


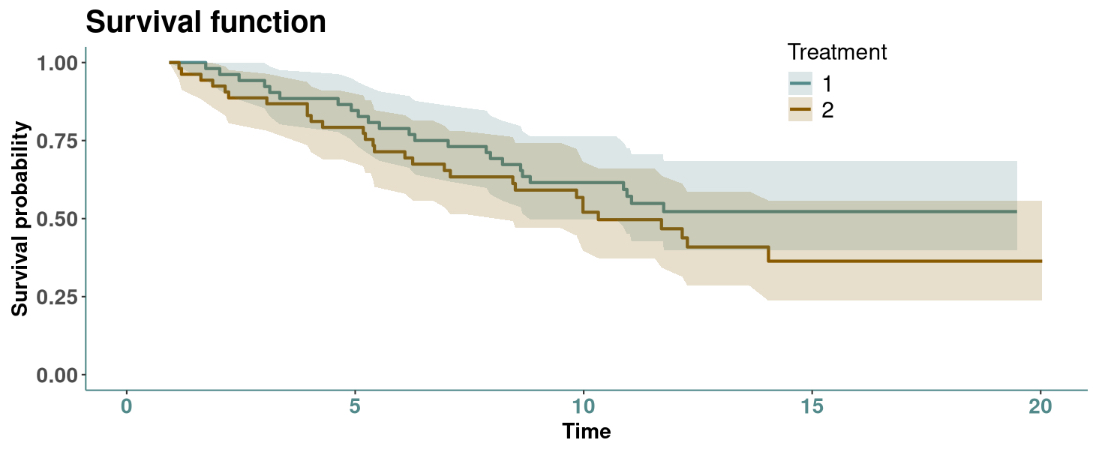


**
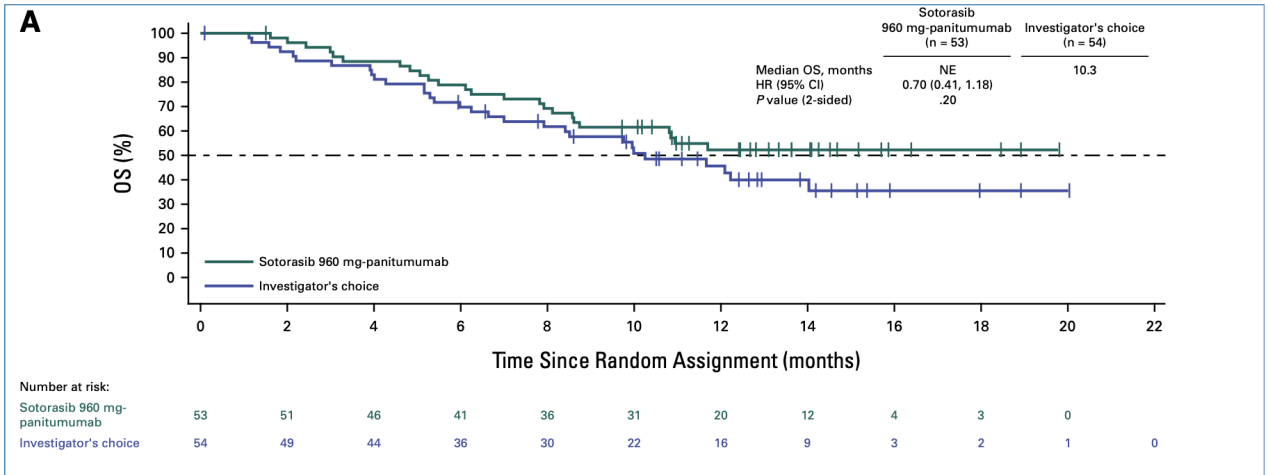
**

**Fakih, 2023-PFS (Sotorasib 240mg)**

**
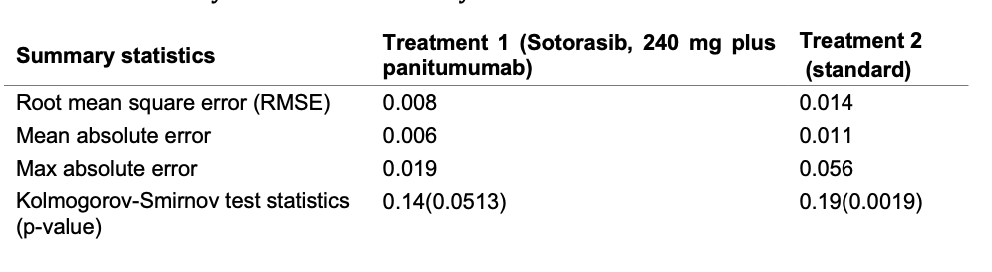
**

**
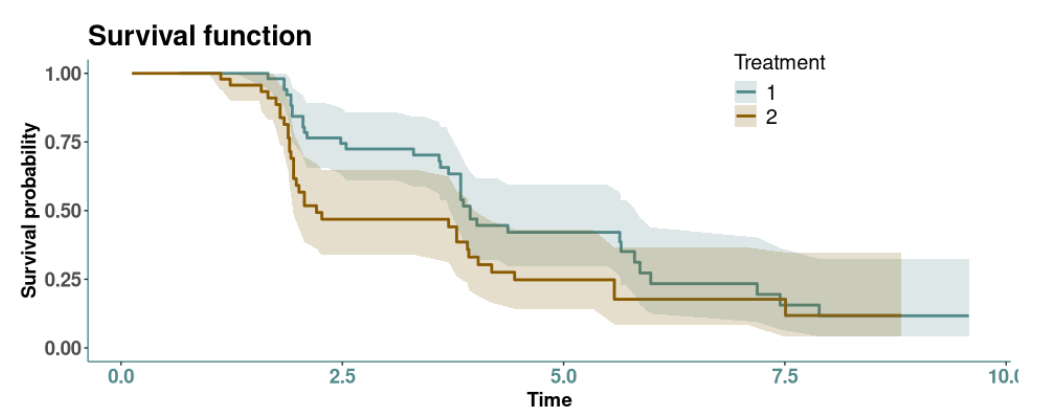
**

**
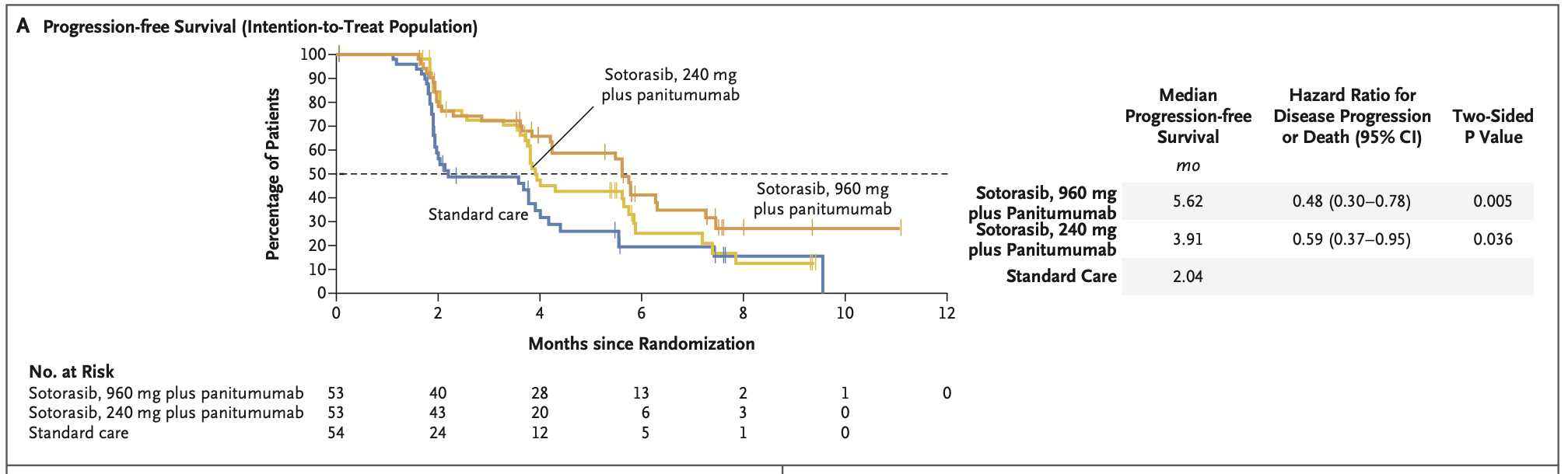
**

**Fakih, 2023-PFS (Sotorasib 960mg)**


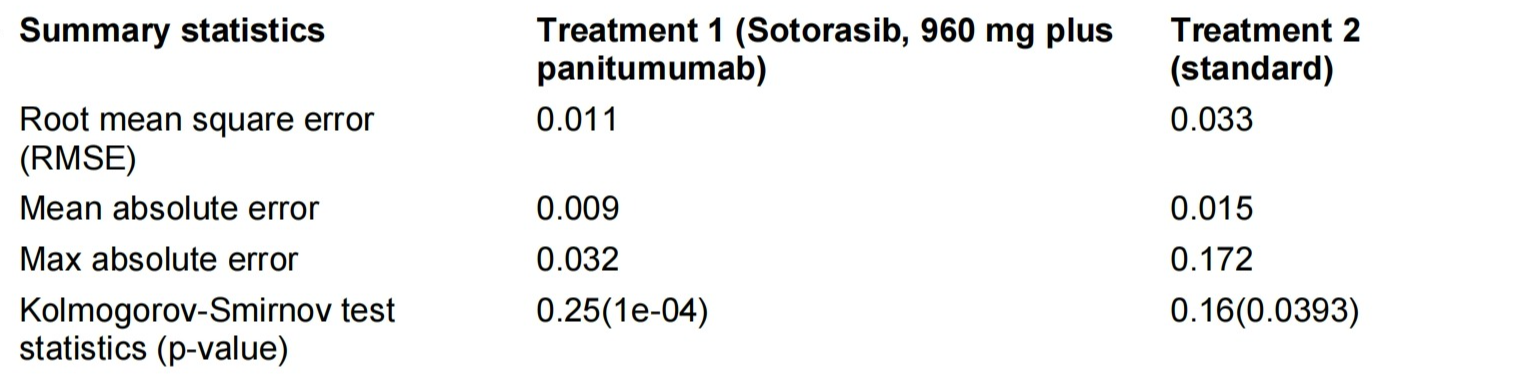


**
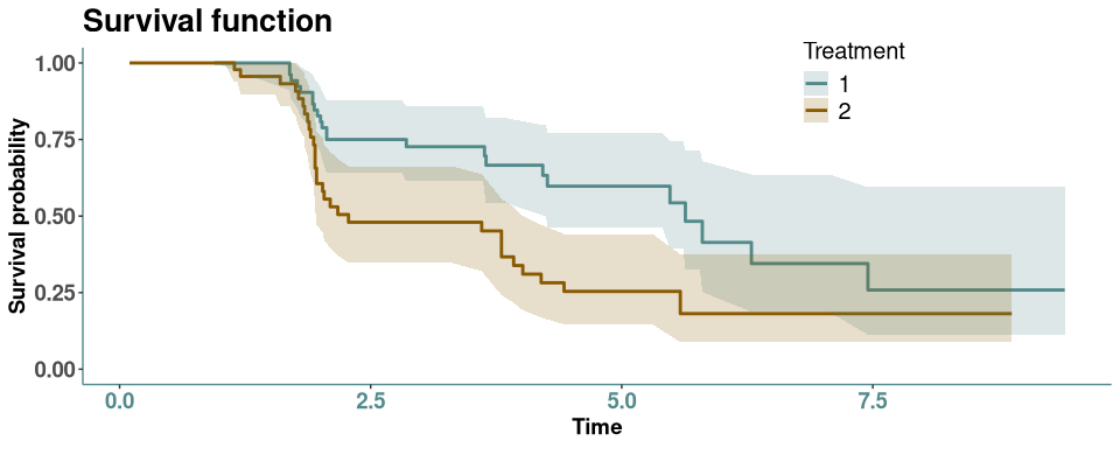
**

**
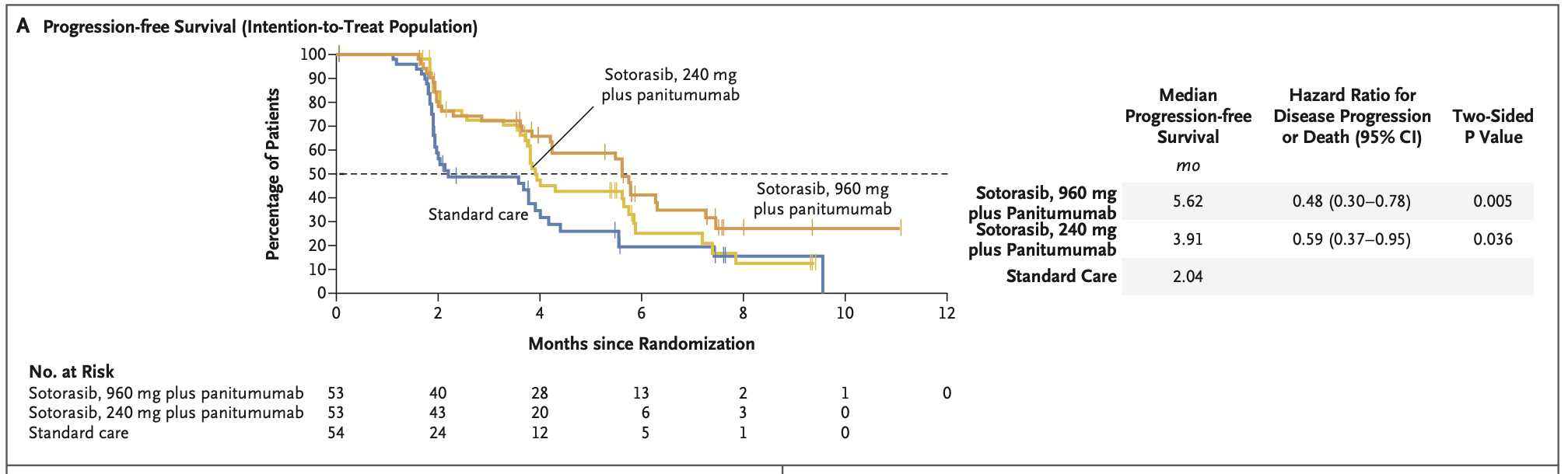
**
